# Comprehensive Bioinformatic and miRNA-Driven Analysis of the Multimeric Canonical I Kappa B Kinase (IKK) Complex in Uterine Corpus Endometrial Carcinoma (UCEC)

**DOI:** 10.3390/biom16091296

**Published:** 2026-09-08

**Authors:** Yasemin Dadas, Enes Karaman, Ergul Bayram, Durmus Ayan

**Affiliations:** 1 Department of Obstetrics and Gynecology, Faculty of Medicine, Nigde Omer Halisdemir University, Nigde 51240, Turkey; 2 Department of Obstetrics and Gynecology, Private Tekden Hospital, Kayseri 38010, Turkey; 3 Medical Biochemistry, Nigde Omer Halisdemir University Research and Training Hospital, Nigde 51100, Turkey; 4 Department of Medical Biochemistry, Faculty of Medicine, Nigde Omer Halisdemir University, Nigde 51240, Turkey; 5 Department of Medical Biochemistry, Dr. Abdurrahman Yurtaslan Ankara Oncology Training and Research Hospital, Ankara 06200, Turkey

**Keywords:** UCEC, IKK complex, IKBKG, NF-κB, CHUK, IKBKB, bioinformatics, prognosis, promoter methylation, miRNA regulation

## Abstract

Uterine corpus endometrial carcinoma (UCEC) is the most common gynecologic malignancy, and aberrant canonical NF-κB signaling is implicated in uterine corpus endometrial carcinoma (UCEC), yet the expression, epigenetic, and prognostic profile of IκB kinase (IKK) complex subunits CHUK (IKKα), IKBKB (IKKβ), and IKBKG (NEMO) remains undefined. This study assessed expression, methylation, miRNA regulation, and clinical relevance of IKK-complex genes in UCEC using public datasets. RNA-seq expression (TCGA-UCEC; GEPIA2) showed significant downregulation of IKBKB in tumors versus normal endometrium (unpaired Wilcoxon), whereas CHUK and IKBKG showed non-significant increases; immunohistochemistry (Human Protein Atlas) illustrated heterogeneity and suggested possible mRNA–protein discordance for IKBKB. Promoter methylation analysis (UALCAN; Illumina 450K) identified CHUK hypomethylation and IKBKG hypermethylation in tumors, with no significant change for IKBKB. Stratification showed IKBKB suppression across clinicopathological strata and TP53 mutant/wild-type tumors; IKBKG decreased across subtypes and stages. Pan-cancer profiling (TIMER2.0) highlighted IKBKB as the broadly dysregulated IKK member across malignancies. STRING networks indicated connectivity with NF-κB mediators (e.g., RELA, NFKB1, TRAF6). miRNA predictions (miRDB/TargetScan) revealed shared regulation (CHUK–IKBKB: 14 miRNAs; CHUK–IKBKG: 2; IKBKB–IKBKG: 0). Kaplan–Meier analysis indicated that elevated IKBKG expression correlated with reduced overall survival (HR = 1.85, *p* = 0.0037), while CHUK expression did not correlate with survival, and IKBKB exhibited a non-significant trend. In a multivariable Cox regression analysis, high IKBKG expression continued to show a significant association with reduced overall survival, even after adjusting for age, histological type, histological grade, and TCGA molecular subtype (adjusted HR = 1.58, 95% CI = 1.00–2.47, *p* = 0.048). These findings collectively suggest the potential prognostic significance of IKBKG in UCEC, although independent validation in larger, comprehensively annotated cohorts is still required. Collectively, these findings suggest IKBKG as a candidate negative prognostic marker and indicator in UCEC, warranting experimental validation.

## 1. Introduction

Endometrial cancer, specifically uterine corpus endometrial carcinoma (UCEC), represents the most prevalent gynecological malignancy, with over 60,000 new cases and more than 10,000 deaths reported annually on a global scale [1]. The morbidity and mortality rates are disproportionately elevated in developing nations [2]. Traditionally, endometrial cancer is classified into two subtypes based on histopathological and clinical characteristics. Type I tumors are generally low-grade, exhibit endometrioid histology, and frequently occur in younger women, with a mean age of 61 years at diagnosis. These tumors are often linked to prolonged exposure to unopposed estrogen and originate from precursor lesions such as endometrial hyperplasia or endometrial intraepithelial neoplasia (EIN) [3,4]. Conversely, Type II or non-endometrioid tumors account for 10–20% of sporadic ECs and are more aggressive, often arising from endometrial intraepithelial carcinoma [4]. Several non-genetic risk factors, particularly pertinent to Type I endometrial cancer, have been identified, including obesity, physical inactivity, exogenous estrogen use, insulin resistance, and tamoxifen therapy in breast cancer patients [5]. Emerging evidence indicates that NF-κB-related signaling pathways play a critical role in endometrial cancer progression; the ERK5/NF-κB axis has been shown to regulate proliferation and survival through NEMO/IKKγ-dependent mechanisms, and inhibition of the AKT/NF-κB pathway via TBK1 suppression has demonstrated significant anti-tumor effects in endometrial cancer models [6,7]. One such pathway is the nuclear factor kappa-light-chain-enhancer of activated B cells (NF-κB) pathway, a critical regulator of inflammation, proliferation, angiogenesis, and cell survival [7]. Immunohistochemical studies have demonstrated increased nuclear NF-κB (p65/RelA) expression in endometrial cancer tissues compared to benign and hyperplastic endometria. Nuclear p65 positivity has been reported in 68% of endometrial carcinomas vs. 12% of normal endometria, with higher expression correlating with advanced stage and poor differentiation. Clinical evidence supports the prognostic significance of NF-κB activation in UCEC. It is reported that nuclear p65 expression was significantly associated with advanced FIGO stage, high tumor grade, deep myometrial invasion, and reduced overall survival in a cohort of 120 endometrial carcinoma patients [8]. Constitutive NF-κB activation has been reported in 72% of endometrioid adenocarcinomas and was associated with resistance to progesterone therapy. These findings suggest that aberrant NF-κB activation is a frequent event in UCEC and may contribute to malignant transformation and therapeutic resistance [9]. Similar associations have been observed in other gynecological malignancies, including ovarian and cervical cancers [6]. The NF-κB pathway can be activated through canonical and non-canonical routes, with the canonical pathway predominantly triggered by external inflammatory stimuli. Central to this activation is the IκB kinase (IKK) complex, which comprises two catalytic subunits—CHUK (IKKα) and IKBKB (IKKβ)—and a regulatory subunit, IKBKG (NEMO) [10,11]. The IKK complex is essential for canonical NF-κB activation in response to inflammatory cytokines, growth factors, and stress signals. Importantly, aberrant IKK activity has been implicated in endometrial cancer. It has been demonstrated that IKBKG (NEMO) is required for ERK5-mediated NF-κB activation and that NEMO depletion significantly reduces proliferation and survival of endometrial cancer cells [6]. Pharmacological inhibition of IKKβ with amlexanox has been shown to suppress AKT/NF-κB signaling and induce apoptosis in endometrial carcinoma cell lines [10]. These findings suggest that the IKK complex is a functionally critical node in UCEC and a potential therapeutic target. Upon activation, the IKK complex phosphorylates inhibitors of NF-κB (IκBs), leading to their degradation and facilitating the nuclear translocation of NF-κB dimers to initiate gene transcription [12]. Beyond its role in NF-κB activation, the IKK complex may contribute to tumorigenesis through NF-κB–independent mechanisms. For instance, CHUK has been implicated in oncogenic signaling downstream of mutant BRAF in colorectal cancer cells [11]. It has been shown in various cancer types that the three subunits of the IKK complex (CHUK, IKBKB, and IKBKG) play distinct and sometimes independent roles in tumor biology. IKBKB (IKKβ) is the main catalytic subunit of the canonical NF-κB pathway and plays a critical role in regulating inflammatory cytokine production and immune response. CHUK (IKKα) can function in both canonical and non-canonical NF-κB pathways and may assume NF-κB-independent roles in epithelial–mesenchymal transition (EMT) and cell invasion. IKBKG (NEMO), as the regulatory subunit, is essential for IKK complex activation and has been reported to play a central role in chemoresistance and tumor cell survival in ovarian and cervical cancers. Although NF-κB pathway activation is known in endometrial cancer, the expression profiles, epigenetic regulation, and prognostic significance of each IKK complex component have not yet been systematically investigated. In light of this information, our study’s hypothesis is that CHUK, IKBKB, and IKBKG genes are differentially regulated in UCEC and that this regulation is associated with tumor progression, clinicopathological features, and patient survival. The specific aims of this study are to characterize the epigenetic profiles of these three genes in UCEC tumor tissues, to correlate expression levels with clinicopathological parameters, to determine their prognostic value through survival analysis, and to map protein–protein interaction networks and microRNA regulatory mechanisms. However, to date, no comprehensive study has systematically investigated the expression and potential role of all IKK complex components in UCEC. It should be noted that the present study is intentionally restricted to the three subunits that form the canonical IKK complex CHUK (IKKα), IKBKB (IKKβ), and IKBKG (NEMO) which function as a stable heterotrimeric unit required for classical NEMO dependent IκBα phosphorylation and canonical NF-κB activation [10,12]. The IKK related kinase IKBKE (IKKε), although implicated in breast cancer as an amplified oncogene [13] and more recently in progestin resistance in UCEC [14], is structurally and functionally distinct: it does not incorporate into the canonical NEMO-containing complex and instead signals primarily through IRF3/IRF7 and non-canonical NF-κB/AKT pathways [15]. IKBKE was therefore excluded from the primary gene set but is acknowledged as an interaction partner in our STRING network analysis. Therefore, this study aims to explore the expression profiles and clinical significance of CHUK, IKBKB, and IKBKG in UCEC using a bioinformatics-based approach.

## 2. Materials and Methods

This study utilized a comprehensive bioinformatics framework to examine the prognostic significance and molecular characteristics of CHUK, IKBKB, and IKBKG in uterine corpus endometrial carcinoma (UCEC). The research did not involve experimental procedures; instead, all analyses were conducted using publicly accessible datasets and online analytical platforms.

### 2.1. Gene Expression Profiling

The expression levels of CHUK, IKBKB, and IKBKG were evaluated utilizing the GEPIA2 platform (http://gepia2.cancer-pku.cn/, accessed on 7 January 2025), which enables extensive transcriptomic comparisons between tumor and normal tissues. GEPIA2 facilitates detailed transcription quantification and subtype-specific assessments [16]. In this study, we analyzed RNA-sequencing data from the TCGA-UCEC cohort (project ID: TCGA-UCEC), consisting of 174 primary tumor samples and 91 normal endometrial tissues, accessed via GEPIA2 on 7 January 2025. Unpaired Wilcoxon rank-sum test was used to account for independent sample structure. To determine whether the expression of canonical IKK complex components differs according to the molecular classification of UCEC, molecular subtype annotations and batch-normalized RNA-seq expression data were sourced from the TCGA UCEC PanCancer Atlas cohort via cBioPortal. The cases were categorized into the four recognized TCGA molecular subtypes: POLE-ultramutated, microsatellite instability (MSI)-hypermutated, copy-number-low (CN-low), and copy-number-high (CN-high). Cases without molecular subtype information were excluded from the analysis. The expression levels of CHUK, IKBKB, and IKBKG were compared across these four molecular subtypes. Given that expression values were analyzed using a non-parametric method, overall differences among groups were evaluated using the Kruskal–Wallis test. If the overall test indicated significance, pairwise comparisons were conducted using Dunn’s post hoc test. *p* values from these pairwise comparisons were adjusted for multiple testing using the Benjamini–Hochberg false discovery rate (FDR) procedure, with an FDR-adjusted *p* value of less than 0.05 considered statistically significant.

### 2.2. Promoter Methylation and Clinical Correlation Analysis

To investigate epigenetic modifications and their clinical associations, the UALCAN web portal (https://ualcan.path.uab.edu/, accessed on 7 January 2025) was utilized. UALCAN facilitates the examination of promoter methylation patterns and gene expression in relation to patient demographics and clinicopathological variables [17]. Promoter methylation data for CHUK, IKBKB, and IKBKG were obtained from TCGA-UCEC via UALCAN (*n* = 438 tumors, *n* = 46 normal tissues), measured using the Illumina HumanMethylation450 BeadChip (Illumina, Inc., San Diego, CA, USA). Beta-values were calculated as the ratio of methylated probe intensity to total signal intensity. Further stratified analyses were performed based on cancer stage, age, race, menopausal status, histological subtype, and body weight using one-way ANOVA for normally distributed data or Kruskal–Wallis test for non-normal distributions, as determined by Shapiro–Wilk test.

### 2.3. Gene Correlation and Mutation-Expression Association Analysis

The expression profiles of CHUK, IKBKB, and IKBKG in tumor tissues and their corresponding normal counterparts were examined utilizing the TIMER 2.0 platform (http://timer.cistrome.org/, accessed on 7 January 2025). The differential expression of these three genes was assessed through the Gene DE module, which employs data sourced from The Cancer Genome Atlas (TCGA). Boxplots were constructed to compare gene expression levels across various tumor types and matched non-tumorous tissues, where available [18]. Statistical analysis was conducted using the Wilcoxon rank-sum test, with *p*-values denoted by asterisks: for * *p* < 0.05, ** for *p* < 0.01, *** for *p* < 0.001. For pan-cancer analyses involving multiple tumor types Benjamini–Hochberg FDR correction at q < 0.05 was applied.

### 2.4. Gene Interaction Network Analysis

To elucidate the functional and physical interaction networks of CHUK, IKBKB, and IKBKG, we employed the STRING database (https://string-db.org/, accessed on 7 January 2025). This resource compiles protein–protein interaction data from diverse sources, including experimental repositories, co-expression analyses, and automated literature mining. Interactions were assessed and scored using confidence metrics and were mapped through orthology-informed networks [19]. In this study, the STRING network was employed not as a discovery tool but as a contextual framework to confirm that CHUK, IKBKB, and IKBKG are functionally embedded within the core NF-κB signaling machinery. To provide disease-specific evidence, we additionally assessed pairwise co-expression correlations among IKK complex genes and key NF-κB pathway mediators (RELA, NFKB1, TRAF6) in the TCGA-UCEC cohort using TIMER 2.0 (purity-adjusted partial Spearman correlation) and GEPIA2 (Pearson correlation on log_2_[TPM + 1] values).

To further analyze the structural organization of the interaction network, a network topology analysis was conducted utilizing the protein–protein associations documented in the STRING-derived network. The network was considered as an undirected graph, with duplicate interactions removed prior to analysis. Metrics such as degree, degree centrality, betweenness centrality, and closeness centrality were computed for each node. Additionally, global network properties, including network density, average clustering coefficient, network diameter, and average shortest path length, were assessed. Network topology metrics were calculated using NetworkX version 3.4.2.

### 2.5. MicroRNA Target Prediction

Potential microRNA regulators of CHUK, IKBKB, and IKBKG were identified using MicroRNA Target Prediction Database (miRDB) (https://mirdb.org/, accessed on 7 January 2025) [20] and TargetScan 8.0 (https://www.targetscan.org/vert_80/, accessed on 7 January 2025) [21,22]. This algorithm predicts miRNA binding sites based on sequence complementarity and evolutionary conservation, aiding in the identification of miRNA-gene regulatory relationships. To substantiate the computationally predicted miRNA–mRNA regulatory interactions through expression data, miRNA-sequencing data from TCGA-UCEC were integrated with the corresponding mRNA expression dataset. This analysis utilized Level 3 Illumina HiSeq miRNA-seq isoform quantification data. As the candidate miRNAs identified via TargetScan Human 8.0 and miRDB were specified at the mature miRNA strand level (e.g., -3p or -5p), mature miRNA expression was extracted from isoform-level data using the corresponding MIMAT accession identifiers, rather than precursor-level miRNA measurements. Expression values for the same mature miRNA were aggregated at the sample level when applicable. TCGA sample identifiers were harmonized between the miRNA and mRNA datasets, and tumor samples from the same patients were matched using TCGA patient identifiers. A total of 329 UCEC tumor cases were successfully matched between the two datasets, with 328 cases having complete expression data available for correlation analysis. Correlations were evaluated exclusively for miRNA–target gene pairs previously predicted by TargetScan Human 8.0 and/or miRDB for CHUK, IKBKB, and IKBKG. Associations between mature miRNA expression and the corresponding target-gene mRNA expression were assessed using two-sided Spearman rank correlation analysis. To account for multiple comparisons across the evaluated miRNA–target pairs, *p* values were adjusted using the Benjamini–Hochberg false discovery rate (FDR) procedure. An FDR-adjusted *p* value of less than 0.05 was considered statistically significant. Given the anticipated inhibitory relationship between miRNAs and their target transcripts, significant inverse correlations were regarded as expression-based support for the predicted regulatory relationships.

### 2.6. Differential Expression Analysis Using GEO Datasets

To further substantiate expression trends, microarray datasets GSE7305 [23] and GSE25628 [24] from the Gene Expression Omnibus (GEO) database were subjected to analysis. These datasets were generated using the Affymetrix Human Genome U133 Plus 2.0 Array platform. Raw CEL files were preprocessed using background correction and quantile normalization via GEO2R default parameters. Differentially expressed genes (DEGs) were identified through GEO2R (https://www.ncbi.nlm.nih.gov/geo/geo2r/, accessed on 7 January 2025), using the limma moderated *t*-test with Benjamini–Hochberg correction for multiple testing. Genes with adjusted *p*-values less than 0.05 and absolute log_2_ fold changes greater than 1 were categorized as significantly upregulated or downregulated.

### 2.7. Independent RNA-Seq Validation and Batch Correction

To strengthen the robustness of our findings, expression of CHUK, IKBKB, and IKBKG was re-examined in independent, more recent RNA-seq cohorts in addition to TCGA-UCEC. The CPTAC-3 UCEC dataset was used as the principal proteogenomic validation cohort [25]. The endometrial cancer cohort GSE119041 served as an external transcriptomic validation set. Where datasets generated on different platforms or in different batches were combined, batch effects were corrected using ComBat-seq for raw counts [26], and, for cross-platform microarray/RNA-seq integration, distributions were harmonized using Training Distribution Matching or quantile normalization [27]; alternatively, the batch term was modeled as a covariate within the limma linear-model framework [28].

### 2.8. Survival Analysis Using Kaplan–Meier Plotter

The prognostic implications of CHUK, IKBKB, and IKBKG gene expression in UCEC were assessed utilizing the Kaplan–Meier Plotter tool (https://kmplot.com/analysis/, accessed on 7 January 2025). Overall survival (OS) was examined by categorizing patients into high and low expression cohorts based on the median expression value of each gene. The platform’s best automated cutoff option was employed to establish thresholds and array quality control was activated to exclude biased datasets. Patients with missing survival data were excluded. Differences in survival between groups were evaluated using the log-rank test, with *p*-values less than 0.05 deemed statistically significant [29]. Furthermore, a Cox proportional hazards regression analysis was conducted to determine whether IKBKG expression independently correlated with overall survival. IKBKG expression was classified into high- and low-expression groups based on the median expression value. Initially, a univariate Cox regression was performed for IKBKG expression. Subsequently, a multivariable Cox proportional hazards model was developed, incorporating IKBKG expression along with age at diagnosis, histological type, histological grade, and TCGA molecular subtype. Histological type was categorized as endometrioid versus non-endometrioid, while histological grade was classified as low grade (G1/G2) vs. high grade (G3/high grade). The TCGA molecular subtype was included as a categorical variable, with the CN-low subgroup serving as the reference category. Hazard ratios (HRs), 95% confidence intervals (CIs), and *p* values were calculated, with *p* < 0.05 considered statistically significant.

## 3. Results

### 3.1. Differential Expression and Methylation Patterns of CHUK, IKBKB, and IKBKG in UCEC

**Figure 1:**
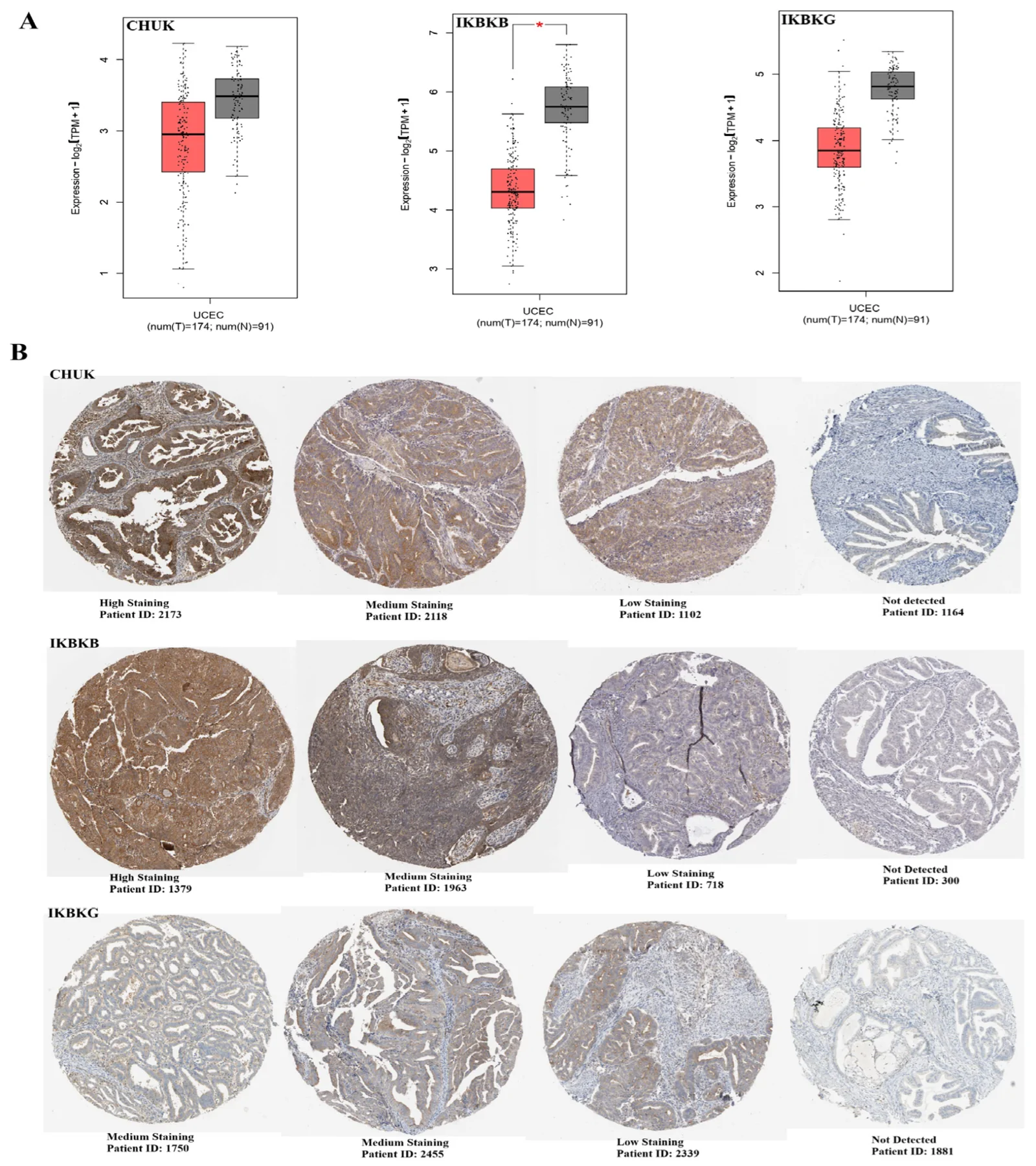
(**A**) mRNA Expression of CHUK, IKBKB, and IKBKG in Uterine Corpus Endometrial Carcinoma. Boxplots show the transcript levels (log_2_[TPM + 1]) of CHUK, IKBKB, and IKBKG in tumor (T, *n* = 174) versus normal (N, *n* = 91) tissues. Asterisks (*) denote statistically significant differences (unpaired Wilcoxon rank-sum test, *p* < 0.05). (**B**) Representative Immunohistochemical Staining Patterns of CHUK, IKBKB, and IKBKG in UCEC Tissues (Human Protein Atlas; qualitative assessment). Representative immunohistochemical staining images obtained from the Human Protein Atlas (HPA version 24.0, accessed 7 January 2025) illustrate staining patterns of CHUK, IKBKB, and IKBKG across UCEC patient samples. Images represent primary endometrial carcinoma surgical specimens. Staining intensity is categorized as high, medium, low, or not detected based on HPA scoring criteria. Patient ID numbers correspond to HPA database identifiers and are indicated below each image. Analysis of promoter methylation using UALCAN revealed that CHUK is hypomethylated in tumor tissues (*p* = 0.000360), whereas IKBKG is hypermethylated in the same tissues (*p* = 0.000395). In contrast, IKBKB displayed relatively stable methylation patterns between normal and tumor samples (*p* = 0.378) (Figure 2).

Transcriptomic profiling utilizing GEPIA2 revealed a significant reduction in the expression of IKBKB (*p* < 0.05) in UCEC tumor tissues compared to normal endometrial controls. In contrast, the expression of CHUK and IKBKG (*p* < 0.05) exhibited a modest yet statistically non-significant increase (Figure 1A). The expression of CHUK protein in UCEC tissues displayed considerable variability, with most samples showing high to medium staining. However, a subset of cases exhibited low or undetectable levels, indicating substantial intertumoral heterogeneity at the protein level. IKBKB protein levels ranged from high to undetectable across patient samples. Although IKBKB mRNA was significantly downregulated in tumor tissues (Panel A), protein staining remained detectable in several cases. However, because HPA immunohistochemistry provides only semi-quantitative, qualitative assessment based on a limited number of representative tissue sections, this observation should be interpreted with caution and does not permit reliable conclusions regarding mRNA–protein discordance or post-transcriptional regulation. IKBKG predominantly exhibited medium to low immunohistochemical staining, with some samples lacking detectable expression. While this pattern appears broadly consistent with the transcriptomic data showing no significant mRNA difference between tumor and normal tissues, the qualitative nature and limited sample coverage of HPA immunohistochemistry preclude definitive assessment of mRNA–protein concordance for IKBKG in UCEC (Figure 1B). The transcriptomic findings were substantiated.

**Figure 3:**
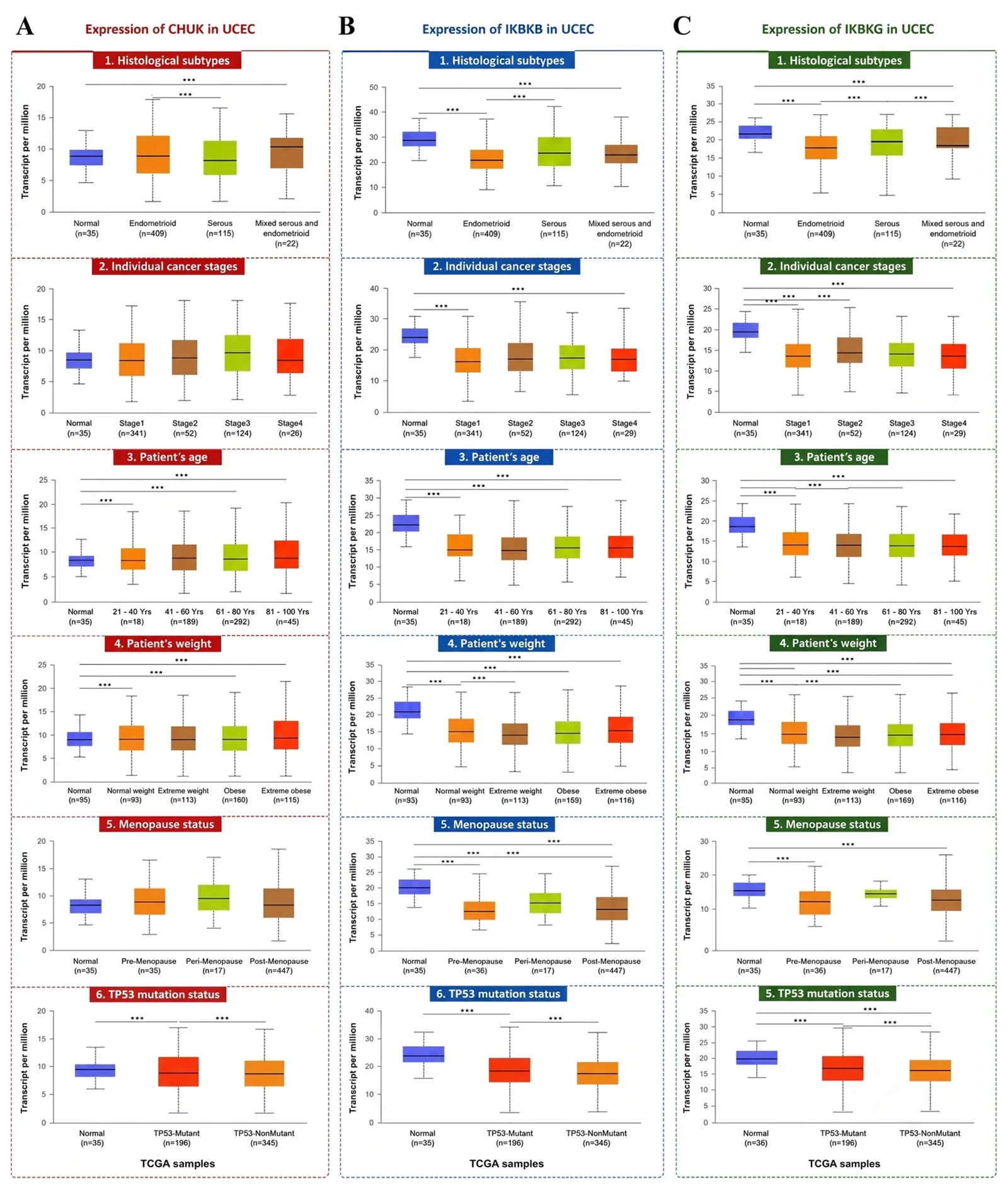
Differential expression of the CHUK gene (**A**), the IKBKB gene (**B**), the IKBKG gene (**C**) in Uterine Corpus Endometrial Carcinoma (UCEC) based on clinicopathological variables. Boxplots illustrate the mRNA expression levels (transcripts per million, TPM) of CHUK, IKBKB, and IKBKG in UCEC samples from TCGA database across various clinical and demographic subgroups: Histological subtypes (endometrioid, serous, mixed, etc.), Cancer stages (Stage I–IV), Patient age groups, Patient weight categories, Menopause status, and TP53 mutation status. Statistical significance between groups was assessed using ANOVA or Kruskal–Wallis tests as appropriate. Box plots show median (line), interquartile range (box) and 5–95 percentile (whiskers), *** *p* < 0.05.

CHUK expression remained relatively stable across all clinicopathological categories, with no significant differences observed between tumors and normal samples. Although a slight reduction was seen in certain subgroups, such as Stage IV and post-menopausal patients, these differences did not reach statistical significance. However, none of these were statistically significant (*p* > 0.05). In weight-related subgroups CHUK expression was found to increase in cancer patients with normal weight (*p* = 3.94 × 10^−2^) compared to normal healthy individuals, while it was found to be decreased in extreme weight patients (*p* = 4.47 × 10^−2^) (Figure 3A).

IKBKB expression was consistently and significantly downregulated in UCEC tumor samples compared to normal tissues across multiple subgroups. IKBKB expressions were markedly downregulated in endometrioid and serous subtypes (*p* = 6.63 × 10^−6^, 4.41 × 10^−4^, respectively). Similarly, IKBKB levels were significantly downregulated in stages I and III compared to normal tissue (*p* = 3.50 × 10^−9^, *p* = 7.20 × 10^−7^, respectively), indicating early and sustained suppression during tumor progression. This trend was further evident in-patient subgroups based on age, with significant downregulation observed in the 21–40, 41–60 and 61–80 age groups (*p* = 2.12 × 10^−5^, *p* = 4.81 × 10^−9^ and *p* = 6.63 × 10^−8^, respectively). Weight-related subgroups (including normal weight, extreme weight, obese, and extremely obese patients) also showed significantly decreased IKBKB expression relative to controls (*p* = 1.71 × 10^−5^, *p* = 2.06 × 10^−5^, *p* = 1.17 × 10^−7^ and *p* = 1.12 × 10^−8^, respectively). Additionally, IKBKB expression was significantly decreased in pre-, peri-, and post-menopausal patients (*p* = 1.55 × 10^−9^, *p* = 2.76 × 10^−2^ and *p* = 9.85 × 10^−8^, respectively), and was suppressed in both TP53-mutant and wild-type (*p* = 5.56 × 10^−3^ and *p* = 1.02 × 10^−10^, respectively) tumors compared to normal samples (Figure 3B).

IKBKG expression was statistically significant decreased in endometrioid and serous histological subtypes (*p* = 2.29 × 10^−6^ and *p* = 4.34 × 10^−2^, respectively) and in all stages (1–4) (*p* = 3.24 × 10^−6^, 3.59 × 10^−2^, 1.05 × 10^−2^ and 6.54 × 10^−3^, respectively) compared to normal tissue. In age-stratified analysis, IKBKG levels were decreased 21–40, 41–60, 61–80 years (6.11 × 10^−3^, 1.96 × 10^−5^ and 9.94 × 10^−5^, respectively), and showed a similar trend in overweight patients and in TP53-mutant tumors (*p* < 0.05) (Figure 3C).

### 3.2. Pan-Cancer Expression Profiling of CHUK, IKBKB, and IKBKG

Analysis of CHUK expression revealed no statistically significant differences between UCEC tumor tissues and their corresponding normal samples, indicating that CHUK may not be actively dysregulated at the transcriptional level in UCEC. Conversely, IKBKB was found to be significantly downregulated in UCEC tumors compared to normal tissue (*p* < 0.001). Given the established role of IKBKB in canonical NF-κB signaling, its decreased transcriptional abundance suggests an alteration in the regulation of this pathway component. However, this does not directly indicate a reduction in NF-κB pathway activity. Additionally, IKBKG expression exhibited no significant difference between tumor and normal tissues in UCEC, implying that IKBKG expression remains relatively stable in this cancer type.

**Figure 4:**
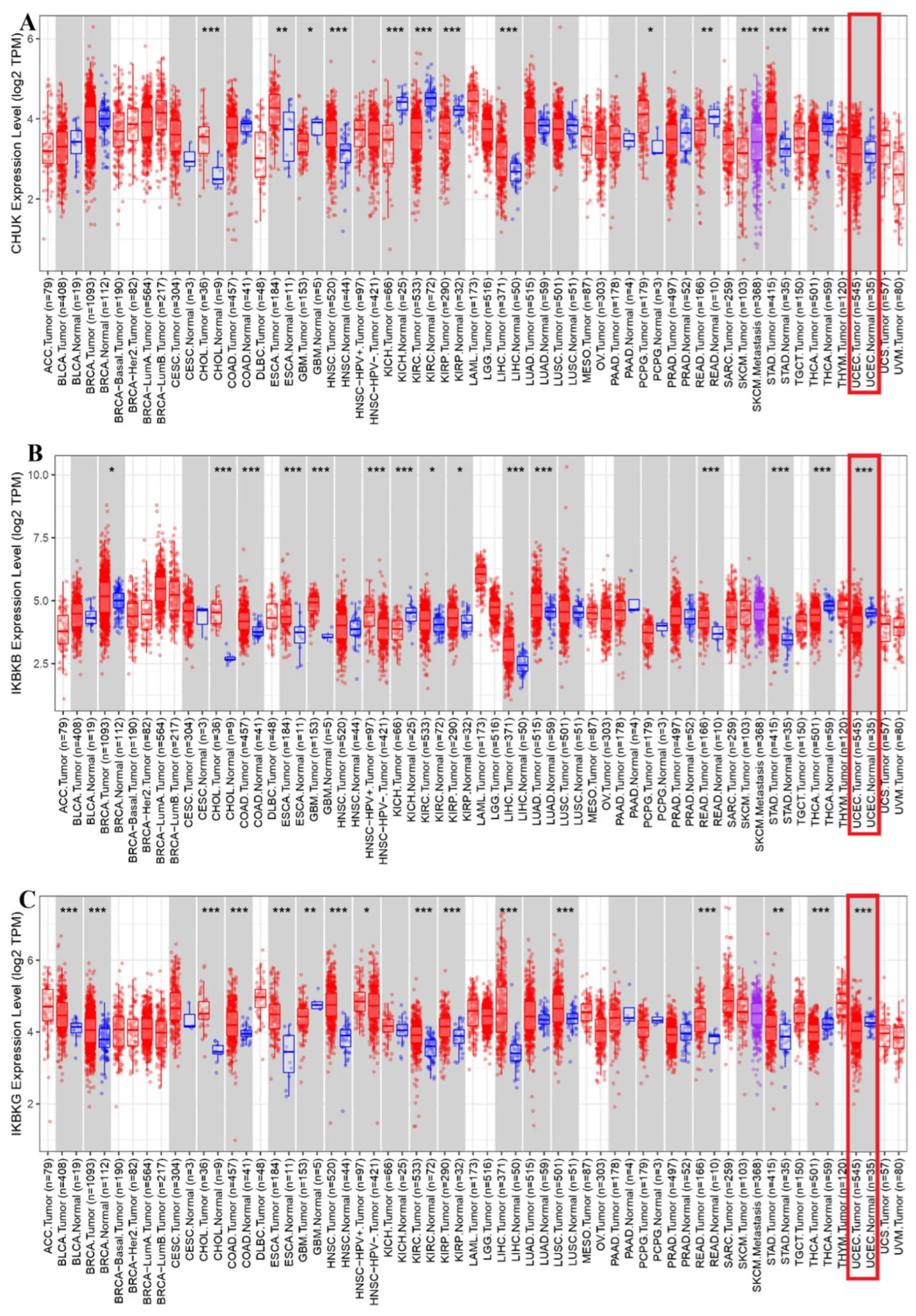
The expression levels of (**A**) the CHUK gene, (**B**) the IKBKB gene, and (**C**) the IKBKG gene in pan-cancer using TIMER2.0, which showed expression in TCGA cancers (red box), the corresponding normal tissues (blue box) and metastasis tissue (purple box); * *p* < 0.05; ** *p* < 0.01; *** *p* < 0.001 (Data for UCEC is marked with red frames).

The pan-cancer expression analysis revealed that CHUK is significantly dysregulated in several malignancies, including CHOL, ESCA, KICH, KIRC, KIRP, LIHC, PCPG, READ, SKCM, STAD, and THCA (Figure 4A). These findings suggest that CHUK may have tumor-type specific roles beyond endometrial cancer. IKBKB showed the most extensive dysregulation among the three genes, with statistically significant expression differences observed in 15 tumor types. These included BRCA, CHOL, COAD, ESCA, GBM, KICH, KIRC, KIRP, LIHC, LUAD, READ, STAD, and THCA (Figure 4B). This widespread alteration suggests that IKBKB may represent a recurrently dysregulated gene across diverse cancer types. IKBKG showed significant dysregulation in eight types of cancer, namely BLCA, BRCA, CHOL, COAD, ESCA, GBM, HNSC, KICH, KIRP, LIHC, LUSC, READ, STAD, and THCA. Although less broadly affected than IKBKB, its recurrent downregulation in gastrointestinal and thoracic cancers points to a potentially conserved role in epithelial tumor biology. In summary, IKBKB appears to be the most consistently dysregulated gene across tumor types, with CHUK and IKBKG also exhibiting significant but more selective alterations. These findings may reflect their unique and overlapping functions in tumorigenesis through NF-κB pathway regulation.

To determine whether expression of the canonical IKK complex components differs according to the molecular classification of UCEC, along with the expression of CHUK, IKBKB, and IKBKG, was assessed across the four TCGA molecular subtypes: POLE-ultra mutated (*n* = 49), MSI-hypermutated (*n* = 148), copy-number-low (CN-low; *n* = 147), and copy-number-high (CN-high; *n* = 163). Significant overall differences among molecular subtypes were identified for CHUK (Kruskal–Wallis *p* = 0.026), IKBKB (*p* = 7.05 × 10^−9^), and IKBKG (*p* = 1.65 × 10^−4^). IKBKB exhibited the most pronounced subtype-dependent variation, with the highest median expression observed in CN-high tumors (944.69), followed by CN-low (824.85), POLE (765.21), and MSI tumors (752.77). After applying the Benjamini–Hochberg correction, IKBKB expression remained significantly elevated in CN-high tumors compared to POLE (FDR = 7.68 × 10^−5^), MSI (FDR = 5.78 × 10^−8^), and CN-low tumors (FDR = 0.0016). IKBKG expression also varied significantly among molecular subtypes, with higher levels in CN-high compared to MSI (FDR = 0.00063) and CN-low tumors (FDR = 0.013), while POLE tumors exhibited higher IKBKG expression than MSI tumors (FDR = 0.0094). For CHUK, the overall subtype effect was less pronounced, with only the CN-low versus CN-high comparison remaining significant after multiple-testing correction (FDR = 0.041). These results indicate that the expression of canonical IKK complex components, particularly IKBKB and IKBKG, is influenced by the molecular subtype of UCEC.

### 3.3. Protein Interaction and Regulatory Network Analysis

**Figure 5:**
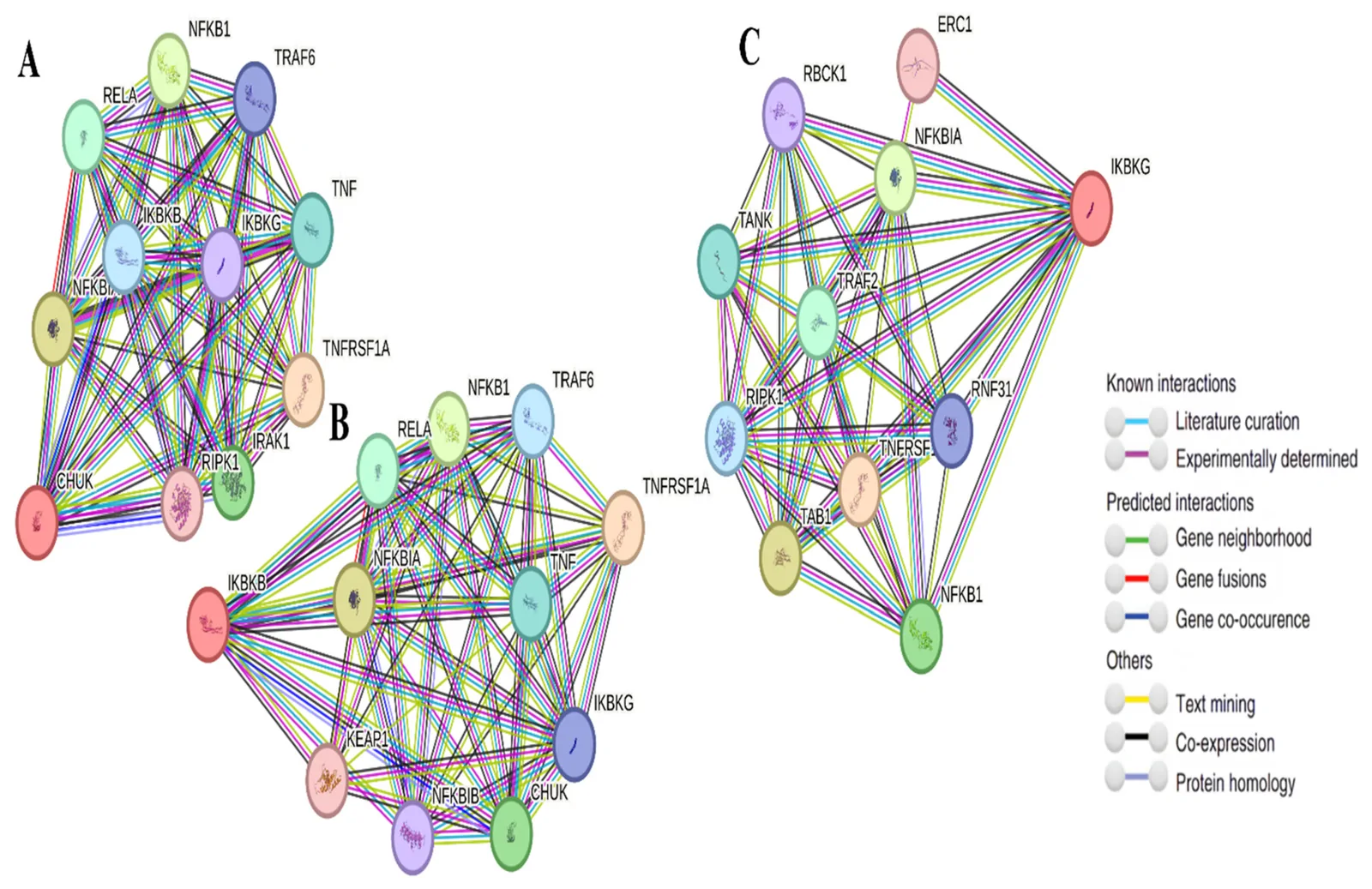
(**A**–**C**) STRING network analysis showing predicted and known protein–protein interactions of CHUK (**A**), IKBKB (**B**), and IKBKG (**C**). Nodes represent proteins, and edges indicate associations based on various sources: experimentally validated (pink), curated databases (blue), co-expression (black), text mining (yellow), and predicted associations including gene neighborhood (green), gene fusions (red), and gene co-occurrence (dark blue). Interaction partners shown include NF-κB pathway components (RELA, NFKB1, NFKBIA), TNF signaling mediators (TNF, TNFRSF1A, TRAF6), and upstream kinases (MAP3K7/TAK1). Combined interaction scores are listed in Table 1.

STRING analysis illustrated strong functional interaction networks among CHUK (Figure 5A), IKBKB (Figure 5B), IKBKG (Figure 5C), and known mediators of the NF-κB pathway, including RELA, NFKB1, and IKBKE. High-confidence scores indicated conserved signaling relationships (Table 1).

STRING analysis revealed a highly interconnected network that links the canonical IKK complex with established components of NF-κB and TNF signaling. To extend the analysis beyond mere visualization of known protein–protein associations, a quantitative network topology analysis was conducted using the interactions detailed in Table 1. The resulting network consisted of 19 nodes and 29 unique edges, exhibiting a network density of 0.170, an average clustering coefficient of 0.399, a network diameter of 3, and an average shortest path length of 2.064. Among the three canonical IKK components, IKBKG demonstrated the highest degree (12), degree centrality (0.667), betweenness centrality (0.595), and closeness centrality (0.750). Both CHUK and IKBKB had a degree of 10 and degree centrality of 0.556, with betweenness centrality values of 0.265 and 0.330, respectively; both exhibited a closeness centrality of 0.692. Therefore, while the identified interaction partners largely represent established components of NF-κB signaling, the quantitative topology analysis highlighted differences in the structural positions of the individual IKK components, with IKBKG occupying the most prominent topological position within the analyzed network.

### 3.4. MicroRNA Target Analysis Result

**Figure 6:**
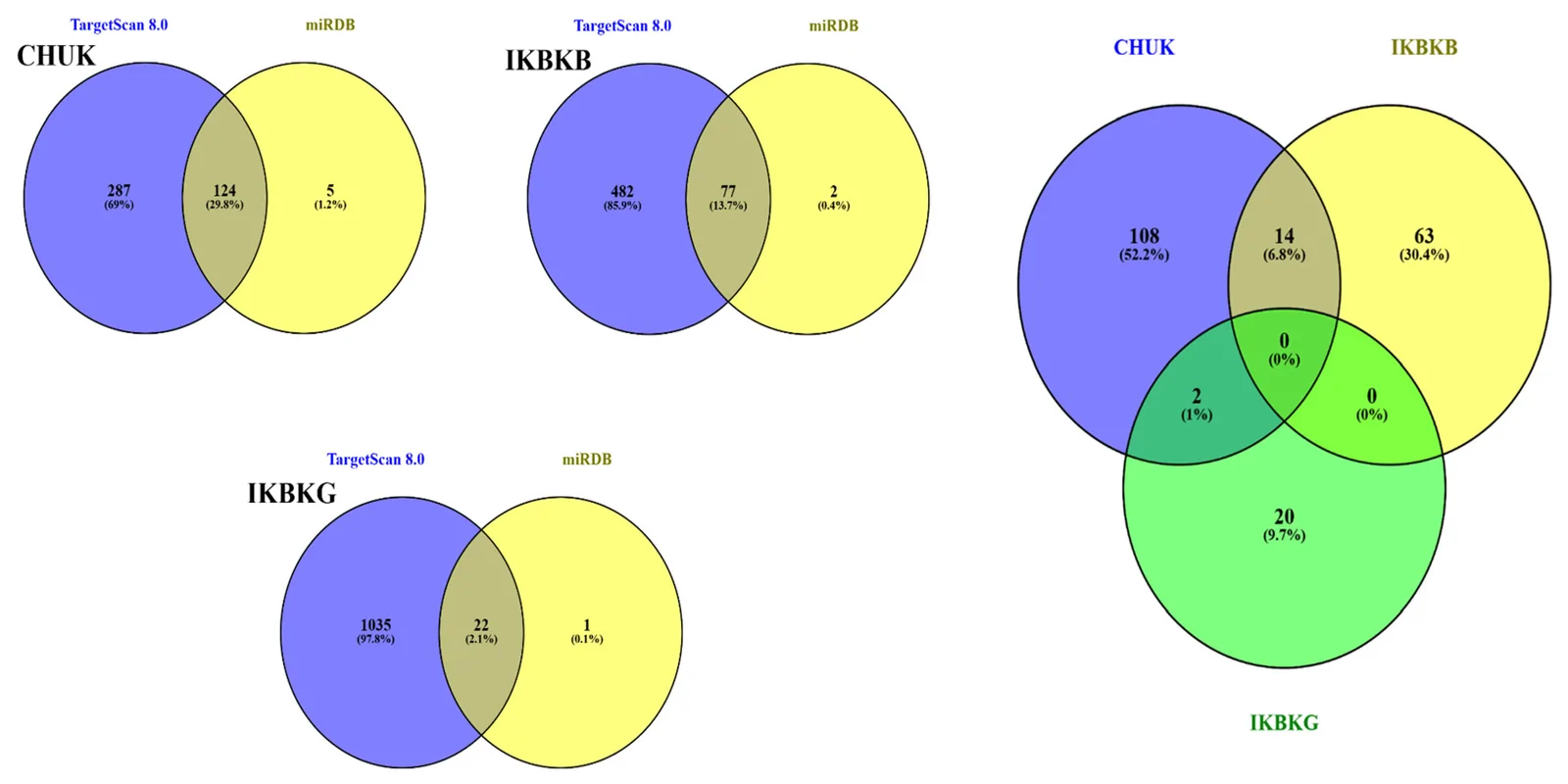
Venn diagram of overlapping numbers of miRNAs that are potential targets of CHUK vs. IKBKB (*n* = 14), CHUK vs. IKBKG (*n* = 2), IKBKB vs. IKBKG (*n* = 0) according to miRDB and TargetScanHuman8.0.

The miRNAs associated with CHUK, IKBKB and IKBKG are listed in Table 2. Accordingly, miRNAs were grouped as a combination of TargetScan Human 8.0 and miRDB databases. (Figure 6). It should be noted that these predictions represent computational hypotheses requiring experimental validation; the biological discussion therefore focuses on candidates with existing functional evidence.

### 3.5. The CHUK and IKBKB Are Affected by the Largest Number of Common miRNAs (n = 14), This Is Followed by the Common miRNAs (n = 2 Impacting CHUK and IKBKG) (Figure 6)

Integration of patient-matched TCGA-UCEC miRNA and mRNA expression data revealed a subset of computationally predicted miRNA–target relationships exhibiting significant inverse expression correlations. Following Benjamini–Hochberg correction for multiple comparisons, three predicted miRNA–mRNA pairs maintained significant inverse correlations. Among the predicted CHUK-targeting miRNAs, hsa-miR-625-5p demonstrated the strongest inverse association with CHUK expression (Spearman ρ = −0.185, *p* = 7.67 × 10^−4^, FDR = 0.0314). Additionally, hsa-miR-23a-3p was inversely correlated with CHUK expression (ρ = −0.174, *p* = 0.00153, FDR = 0.0325). Regarding IKBKB, hsa-miR-148a-3p exhibited a significant inverse correlation with IKBKB mRNA expression (ρ = −0.174, *p* = 0.00159, FDR = 0.0325). Conversely, none of the evaluated predicted IKBKG-targeting miRNAs showed a significant inverse correlation with IKBKG expression after FDR correction. Although the observed correlations were modest in magnitude, the direction and statistical significance of these associations provide expression-based support for the predicted hsa-miR-625-5p–CHUK, hsa-miR-23a-3p–CHUK, and hsa-miR-148a-3p–IKBKB relationships in UCEC. These associations should, however, be interpreted as supportive evidence rather than confirmation of direct miRNA-mediated regulation.

### 3.6. Validation in GEO Datasets

**Figure 7:**
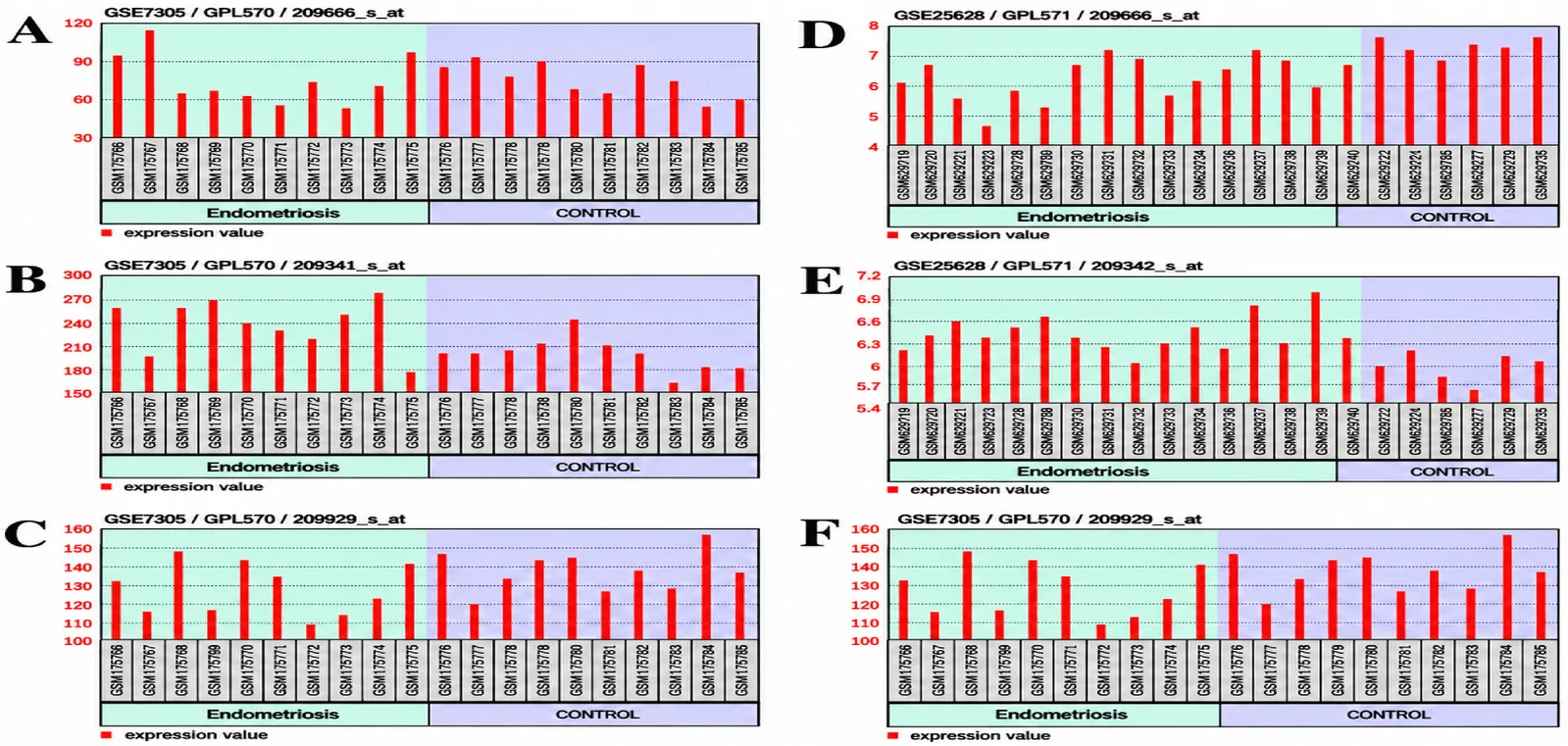
External validation of IKK complex subunits expression using GEO datasets. Expression levels of CHUK, IKBKB, and IKBKG were validated in two independent datasets. Upper panels represent results from the GSE7305 dataset for (**A**) CHUK, (**B**) IKBKB, and (**C**) IKBKG. Lower panels represent results from the GSE25628 dataset for (**D**) CHUK, (**E**) IKBKB, and (**F**) IKBKG. Red bars indicate individual sample expression values within UCEC (green background) and control (purple background) groups. Statistical significance was determined using the GEO2R tool.

To provide a broader molecular context, IKK complex gene expression was also examined in two publicly available endometriosis-related GEO datasets. GSE7305 comprises endometriosis versus normal endometrium samples (*n* = 20; GPL570) [23] and GSE25628 contains ectopic endometrium, eutopic endometrium from women with endometriosis, and normal-donor endometrium samples (*n* = 22; GPL571) [24]. In GSE7305, IKBKB was modestly but significantly upregulated in disease tissue (adjusted *p* = 0.0357, log_2_FC = +0.23), a direction opposite to its downregulation in the TCGA-UCEC analysis. CHUK and IKBKG showed no significant changes (adjusted *p* = 0.924, log_2_FC = −0.02; adjusted *p* = 0.281, log_2_FC = −0.104, respectively). In GSE25628, CHUK was significantly downregulated (adjusted *p* = 0.00831, log_2_FC = −1.05), IKBKG was significantly upregulated (adjusted *p* = 0.00771, log_2_FC = +0.45), and IKBKB did not reach significance (adjusted *p* = 0.112, log_2_FC = −0.32) (Figure 7).

### 3.7. Survival Analysis Results Using Kaplan–Meier Plotter

**Figure 8:**
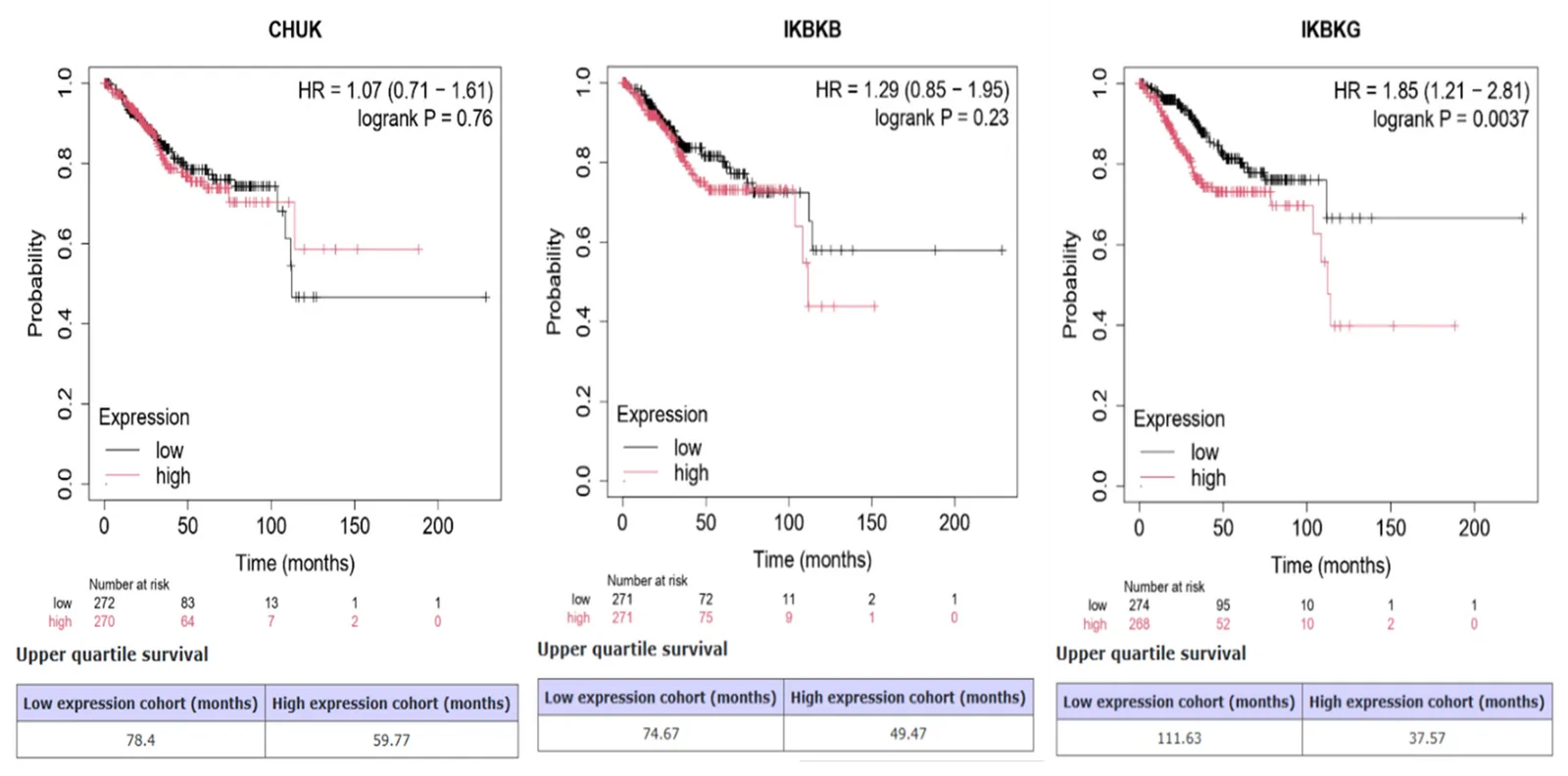
Kaplan–Meier survival curves evaluating the prognostic significance of IKK complex gene expression (CHUK, IKBKB, and IKBKG) in Uterine Corpus Endometrial Carcinoma (UCEC).

There is no statistically significant difference in overall survival between high and low CHUK expression groups (*p* = 0.76). The HR is close to 1 and the confidence interval crosses 1, suggesting no strong prognostic value of CHUK expression in this cohort. Although there is a trend toward worse survival with higher IKBKB expression, it is not statistically significant (*p* = 0.23). The HR indicates a 29% increased risk of death with high expression, but the CI includes 1. Thus, no definitive conclusion can be drawn about its prognostic relevance. IKBKG shows a statistically significant association with poor overall survival (*p* = 0.0037). Patients with high IKBKG expression had markedly shorter survival (37.57 vs. 111.63 months), and the HR of 1.85 indicates an 85% higher risk of death in the high expression group. This is consistent with IKBKG being a candidate negative prognostic indicator in this cancer cohort negative prognostic biomarker in this cancer cohort (Figure 8).

To assess the prognostic significance of IKBKG, a Cox proportional hazards regression analysis was conducted. The univariate analysis revealed that elevated IKBKG expression correlated with reduced overall survival (HR = 1.62, 95% CI 1.04–2.52, *p* = 0.034). Upon adjusting for age, histological type, histological grade, and TCGA molecular subtype, high IKBKG expression continued to show a significant association with decreased overall survival (adjusted HR = 1.58, 95% CI 1.00–2.47, *p* = 0.048). Additionally, a high histological grade was independently linked to an increased risk of mortality (HR = 3.17, 95% CI 1.54–6.53, *p* = 0.0018), while the POLE molecular subtype was associated with a reduced mortality risk compared to the CN-low reference group (HR = 0.19, 95% CI 0.04–0.90, *p* = 0.036). These results underscore an independent association between increased IKBKG expression and adverse overall survival, as demonstrated by the variables included in the multivariable model (Table 3).

## 4. Discussion

The present study conducts a comprehensive bioinformatics analysis of the IκB kinase (IKK) complex—comprising CHUK (IKKα), IKBKB (IKKβ), and IKBKG (NEMO)—in uterine corpus endometrial carcinoma (UCEC). The canonical NF-κB pathway, facilitated by this complex, has been extensively associated with inflammation-related tumorigenesis; however, its specific role in endometrial cancer remains insufficiently investigated. Our findings characterize transcriptional dysregulation, prognostic relevance, protein expression levels, molecular interactions, and miRNA regulatory networks of IKK genes, providing an initial computational characterization of their potential function in UCEC.

Gene expression analysis indicated a significant upregulation of all three IKK complex members in UCEC tissues compared to normal endometrial controls. Whether these mRNA-level alterations are accompanied by corresponding changes in IKK protein abundance, kinase activity, or downstream NF-κB transcriptional output remains to be determined. Notably, despite the overall upregulation of the NF-κB pathway, IKBKB was significantly downregulated, suggesting a distinct regulatory mechanism or feedback control. Furthermore, IKBKG expression was downregulated in advanced-stage tumors and high-grade histological subtypes. A pan-cancer overview was included to place the UCEC findings within the broader landscape of human malignancies. Rather than serving as a separate analytical objective, this comparison was intended to determine whether CHUK, IKBKB, and IKBKG exhibit a universal pattern of dysregulation or displays tumor-specific expression characteristics. The observed heterogeneity across different cancer types suggests that CHUK, IKBKB, and IKBKG are regulated in a tissue-dependent manner, supporting the notion that its biological significance in UCEC should be interpreted within the unique molecular and cellular environment of the endometrium. Such pan-cancer comparisons are increasingly incorporated into integrative bioinformatics studies to provide biological context and improve interpretation of cancer-specific findings. In line with this approach, our pan-cancer expression analysis (Figure 4) underscores the distinct regulatory landscape of the IKK complex in UCEC compared to other malignancies. The observed downregulation of IKBKG (also known as NEMO, NF-κB essential modulator) in advanced-stage tumors and high-grade histological subtypes may appear paradoxical, given its well-established role in the canonical NF-κB signaling pathway, which typically supports tumor progression by promoting inflammation, proliferation, and resistance to apoptosis [30,31]. Recent research suggests that the role of IKBKG in tumor biology is highly dependent on the specific context, with its expression potentially varying across different tumor stages, subtypes, and microenvironmental conditions [32,33].

In aggressive and poorly differentiated tumors, the downregulation of IKBKG expression could hypothetically reflect a biological transition from canonical NF-κB signaling to alternative survival pathways, such as the non-canonical NF-κB (RELB/p52 axis), STAT3, or MAPK pathways [34,35]; however, this hypothesis remains to be tested experimentally. It is tempting to speculate that this may represent an adaptive mechanism through which tumor cells sustain growth and survival via IKBKG-independent pathways, although direct evidence for such a pathway switch in UCEC is currently lacking.

Moreover, epigenetic modifications, such as promoter hypermethylation, or genomic alterations, including deletions or mutations affecting IKBKG, may contribute to its silencing in high-grade tumors [36]. Tumors characterized by high genetic instability frequently demonstrate extensive deregulation of regulatory genes, including those implicated in immune and inflammatory signaling pathways. Notably, the downregulation of IKBKG has been hypothesized to be linked to mechanisms of immune evasion in other contexts. Although NF-κB activation is typically oncogenic, it can paradoxically facilitate immune surveillance by augmenting the expression of pro-inflammatory cytokines and chemokines [37]. Based on this rationale, it is conceivable that the reduction in IKBKG expression could diminish immune activation; however, whether this translates into immune evasion in UCEC requires validation through immune cell infiltration and functional immune profiling studies.

The reduced expression of IKBKG in late-stage and high-grade tumors may suggest a tumor-driven mechanism to diminish immune detection and leverage alternative oncogenic pathways. These findings are consistent with, but do not definitively establish, the hypothesis of a dual role for IKBKG, which may function as a tumor promoter in early stages and potentially as a tumor suppressor or immunogenic modulator in advanced disease. Functional studies are needed to confirm this stage-dependent model. Further research is necessary to fully elucidate the role of IKBKG in tumor progression. With respect to therapeutic targeting, no direct NEMO inhibitor has been tested in UCEC models to date. The most disease-relevant functional evidence comes from the ERK5–NEMO–NF-κB axis, where ERK5 inhibition or depletion reduced NEMO levels and NF-κB/p65 activity, increased apoptosis, and enhanced chemosensitivity in endometrial cancer cells and xenografts [6]. In parallel, preclinical studies in non-UCEC systems have explored direct NEMO-targeting approaches, including peptides that disrupt the NEMO-binding domain (NBD) on IKKβ and small molecules that block the IKKβ–NEMO protein–protein interaction. However, these strategies remain at the proof-of-concept stage and have not been validated in endometrial cancer. Given the essential role of NEMO in normal immune signaling, any NEMO-directed strategy would need to demonstrate an adequate therapeutic window between tumor cells and the host immune system. Accordingly, IKBKG should be regarded as a candidate warranting experimental validation in biomarker-stratified UCEC models rather than a clinically actionable therapeutic target.

The pharmacological landscape of NEMO-directed strategies includes NBD-based peptides, IKKβ–NEMO interaction disruptors, and NEMO–ubiquitin binding inhibitors, all of which remain at the proof-of-concept stage in non-UCEC models. In UCEC, the ERK5–NEMO–NF-κB axis represents the most advanced preclinical rationale, as ERK5 inhibition indirectly reduced NEMO-dependent NF-κB signaling and synergized with paclitaxel in xenograft models [6]. This indirect approach may offer a more tractable strategy while avoiding the immunosuppressive consequences of systemic NEMO blockade. Importantly, patient selection should not rely solely on IKBKG mRNA levels; biomarker-stratified approaches incorporating NEMO protein quantification, phospho-IKK, phospho-p65, and NF-κB transcriptional output assessment will be essential for identifying responsive tumors.

The comprehensive transcriptomic analysis revealed a consistent and statistically significant downregulation of IKBKB expression in UCEC tumor samples compared to normal endometrial tissues across multiple independent clinical subgroups. Within the TCGA-UCEC cohort, this observation was internally reproducible across multiple clinicopathological subgroups, supporting its robustness as a cohort-level finding. Notably, IKBKB expression was markedly suppressed in both endometrioid and serous subtypes, as well as in early (Stage I) and advanced (Stage III) stages, suggesting that IKBKB repression is not only an early molecular event but also sustained during tumor progression. Furthermore, this trend persisted across diverse patient demographics, including age (21–80 years), weight categories (normal to extremely obese), and menopausal status (pre, peri-, and post-menopausal), indicating that IKBKB suppression is largely independent of hormonal or metabolic influences and may represent a core feature of UCEC pathophysiology.

The observed bulk-tissue downregulation of IKBKB in UCEC could reflect a tumor-cell-intrinsic mechanism limiting NF-κB-mediated expression of pro-inflammatory cytokines and antigen-presenting molecules; however, because the TCGA-UCEC RNA-seq data represent a mixture of malignant, immune, and stromal cells, the contribution of altered cellular composition to this expression difference cannot be excluded without purity-adjusted or deconvolution-based analyses [37]. although this putative immune evasion mechanism has not been directly demonstrated in endometrial carcinoma. The observation that IKBKB suppression was evident in both TP53-mutant and wild-type tumors is noteworthy and may suggest a shared regulatory alteration, though the underlying mechanism remains to be elucidated.

Furthermore, it is plausible that the persistent downregulation of IKBKB could result in a compensatory transition towards alternative oncogenic signaling pathways, such as non-canonical NF-κB signaling, PI3K/AKT, or IL-6/STAT3 axes, as has been reported in other malignancies [34,38]. Whether such pathway switching occurs in UCEC warrants experimental investigation. Epigenetic dysregulation is an established feature of endometrial carcinogenesis and may affect gene expression through promoter-associated CpG methylation as well as alterations in distal regulatory elements and chromatin organization [39,40,41]. In the present study, UALCAN-based promoter methylation analysis revealed gene-specific patterns: CHUK was significantly hypomethylated in tumors with a non-significant mRNA increase, IKBKB showed no significant promoter methylation difference despite significant mRNA downregulation, and IKBKG was significantly hypermethylated with a nonsignificant mRNA increase. It should be noted that these methylation and expression results represent independent tumor versus normal group comparisons conducted on nonidentical sample sets and do not constitute within-sample methylation–expression correlations. Accordingly, a direct regulatory relationship between promoter methylation and transcript levels cannot be inferred from these findings alone. The unchanged aggregate promoter methylation of IKBKB does not support promoter hypermethylation as the principal mechanism underlying its reduced transcript abundance; alternative explanations, including regulation at distal enhancer elements, chromatin remodeling, copy-number alterations, or post-transcriptional mechanisms, remain to be investigated. Similarly, the observation that IKBKG promoter hypermethylation was not accompanied by a corresponding decrease in mRNA levels suggests that a simple one-directional promoter-silencing model is insufficient to account for the observed expression patterns, and that other regulatory layers may modulate the transcriptional output independently of aggregate promoter methylation status. These observations underscore the gene- and region-specific nature of methylation alterations in UCEC and highlight the need for future probe-level, matched-sample correlation studies to determine whether the observed methylation differences are functionally linked to transcriptional regulation.

Collectively, these bulk-tissue findings suggest that IKBKB expression is consistently lower in UCEC across multiple clinicopathological subgroups; however, persistence across clinical strata does not establish independence from cellular composition, as those strata may themselves differ in tumor purity and immune/stromal abundance. Therefore, IKBKB’s candidacy as a computational prognostic indicator remains contingent on confirmation through purity-adjusted analyses or cell-type-resolved expression data.

Molecular subtype analysis further revealed that the expression patterns of canonical IKK complex components vary across UCEC. The most notable subtype-dependent variation was identified for IKBKB, which exhibited significantly higher expression in CN-high tumors compared to POLE-ultramutated, MSI-hypermutated, and CN-low tumors, following correction for multiple comparisons. IKBKG also demonstrated significant molecular subtype-associated variation, with elevated expression in CN-high tumors relative to MSI and CN-low tumors, whereas CHUK displayed a comparatively weaker subtype-dependent pattern. These findings are particularly significant as the TCGA molecular classification captures major biological differences among UCEC tumors that are not fully represented by conventional histopathological stratification. The enrichment of IKBKB and IKBKG expression in the CN-high subgroup suggests that alterations in canonical IKK/NF-κB signaling may vary according to the molecular context of UCEC and may be particularly pertinent to tumors with a copy-number-high phenotype. However, these expression-based associations do not confirm subtype-specific pathway activation or functional dependency and thus require validation in molecularly annotated experimental and clinical cohorts.

The inclusion of independent GEO datasets provides valuable cross-disease molecular context for IKK complex gene expression. However, several factors should be considered when interpreting the observed discrepancies between the TCGA-UCEC and GEO analyses. First, GSE7305 and GSE25628 are endometriosis cohorts rather than endometrial carcinoma series; consequently, the biological contrast in these datasets (endometriosis versus normal endometrium) differs fundamentally from the TCGA-UCEC tumor-versus-normal comparison. Endometriosis is a benign, estrogen-dependent inflammatory condition with a distinct molecular landscape, and a gene may exhibit divergent expression behavior across these two pathobiological contexts without contradiction. This biological non-equivalence is likely the primary contributor to the observed directional discordance, particularly for IKBKB, which was upregulated in the GSE7305 endometriosis comparison but consistently downregulated across UCEC subgroups in TCGA. Second, RNA-seq and Affymetrix microarray platforms differ in their fundamental measurement principles: microarrays are hybridization-based and possess a limited dynamic range (typically 100- to 1000-fold), rendering them susceptible to signal saturation and background noise, whereas RNA-seq is a digital [42,43,44,45,46], count-based method with a dynamic range exceeding 10,000-fold and superior sensitivity for lowly expressed transcripts [47,48]. Because microarray probes cannot always distinguish between splice isoforms and are susceptible to cross-hybridization, discordance between platforms tends to be greatest for low-abundance or alternatively spliced signaling genes such as the IKK complex members [49]. Third, differences in sample size and statistical power (20 samples in GSE7305, 22 in GSE25628, versus hundreds in TCGA-UCEC) can influence the detection and direction of modest expression changes. Furthermore, integrating heterogeneous cohorts introduces batch effects—systematic non-biological variation that can mimic or mask true differences, particularly when confounded with the biological variable of interest [50]. Because biological condition, platform, and cohort structure change simultaneously across these comparisons, the contribution of each factor cannot be isolated without a controlled cross-platform study.

Despite these considerations, the cross-disease analysis is informative. The divergent IKBKB behavior between endometriosis and UCEC may itself carry biological significance, potentially reflecting disease-specific regulatory programs within the NF-κB signaling axis. Within the TCGA-UCEC cohort, IKBKB downregulation was consistently observed across histological subtypes, disease stages, age groups, weight categories, and menopausal status, supporting its robustness as an internal finding. Consistent with published observations that cross-platform concordance for differentially expressed genes in endometrial cancer is approximately 77% [51] we interpret the partial discordance as a combined consequence of biological and technical heterogeneity rather than a refutation of the UCEC-specific findings. Importantly, the observed transcript-level variability does not contradict the functional activation of the IKK/NF-κB axis, which is further supported by phosphoproteomic (NF-κB-p65_pS536) and functional evidence [6,25]. Nevertheless, independent validation in a dedicated endometrial carcinoma cohort with a comparable tumor-versus-normal design remains warranted and is acknowledged as a limitation of the present study.

The prognostic significance of these findings was further assessed using Kaplan–Meier survival analysis. Although the expression levels of CHUK and IKBKB did not exhibit a significant correlation with overall survival, increased expression of IKBKG was associated with a poor prognosis. This observation is consistent with its clinicopathological distribution and suggests a role in disease progression, although a direct causal relationship and any association with therapeutic resistance remain to be established through functional studies. These results corroborate previous studies in ovarian and cervical cancers, which identified NEMO/IKBKG as a contributor to tumor cell survival, chemoresistance, and immune evasion [6,52,53]. The association between elevated IKBKG expression and poorer overall survival remained significant after adjusting for age, histological type, histological grade, and TCGA molecular subtype in a multivariable Cox regression analysis. High IKBKG expression correlated with an approximately 1.6-fold increase in mortality risk, indicating its potential prognostic significance beyond these clinicopathological and molecular factors. However, the clinical dataset available did not allow for the inclusion of all potentially relevant prognostic covariates, particularly the overall disease stage. The borderline statistical significance of the adjusted IKBKG association necessitates cautious interpretation. Therefore, validation in independent, comprehensively annotated clinical cohorts is essential before IKBKG can be considered an established independent prognostic biomarker.

As qualitative supporting evidence, immunohistochemical images obtained from the HPA showed cytoplasmic and occasional nuclear staining of IKBKG in a subset of UCEC samples, with comparatively weaker staining in the limited normal endometrial sections available. This cytoplasmic pattern is broadly consistent with the known scaffolding role of IKBKG in proximal NF-κB signaling, and the occasional nuclear staining may reflect reported co-activator functions. However, it must be emphasized that HPA-derived IHC data are based on a small number of representative tissue sections with semi-quantitative scoring and cannot reliably quantify protein abundance or serve as independent validation of the transcriptomic findings. Tissue heterogeneity, fixation variability, and anti- body-dependent staining differences further limit the interpretive scope of these observations.

To elucidate the broader functional implications of IKK gene dysregulation, we performed a STRING-based protein–protein interaction (PPI) network analysis. Although the STRING-derived interaction network reflects general protein–protein associations rather than UCEC-specific experimental data, the co-expression correlations observed in the TCGA-UCEC transcriptome and the elevated phospho-p65 abundance in the CPTAC/TCGA proteome collectively suggest that these interactions are operationally relevant in endometrial carcinoma. The STRING network therefore serves as a validated pathway scaffold onto which our UCEC-specific expression, methylation, and survival findings are mapped. This analysis uncovered extensive interactions between the IKK complex and several key signaling molecules, including RELA (p65), NFKBIA, TRAF6, MAP3K7 (TAK1), and TNFRSF1AAlthough numerous STRING-derived interaction partners are recognized components of the NF-κB pathway, the network topology analysis yielded additional quantitative insights into the relative structural positions of the three canonical IKK components. IKBKG demonstrated the highest values in degree, degree centrality, betweenness centrality, and closeness centrality. Notably, its elevated betweenness centrality indicates a more prominent bridging role within the analyzed network, in contrast to CHUK and IKBKB, which, despite their high connectivity, exhibited lower betweenness centrality. These results quantitatively differentiate the network position of IKBKG from those of the other canonical IKK components. However, as these topology metrics are derived from a knowledge-based STRING interaction network, they should be regarded as indicators of network structure rather than as evidence of UCEC-specific functional dependency. The PPI map positions the IKK complex as a central regulatory hub for immune response, cell proliferation, and apoptosis evasion. Notably, IKBKG emerged as a primary interaction node, linking NF-κB signaling to additional oncogenic pathways such as TNF, IL-1, and TLR cascades. This extensive connectivity may underscore its stronger phenotypic associations in UCEC, distinguishing it from CHUK and IKBKB.

Furthermore, miRNA–target predictions have identified several tumor-associated miRNAs that may regulate the IKK complex, including miR-340-5p and miR-23a-3p, which are reportedly downregulated in endometrial and other gynecologic malignancies [54,55]. The dysregulation of these miRNAs may lead to changes in the transcriptional regulation of IKK complex components. However, the functional implications for NF-κB pathway activity cannot be determined solely from expression data. For example, miR-23a-3p is known to influence epithelial-to-mesenchymal transition (EMT), invasion, and chemoresistance, all of which are characteristic of advanced UCEC [54]. The loss of such regulatory miRNAs may therefore act synergistically with genetic and epigenetic alterations to enhance the oncogenic effects of IKK signaling.

Among the predicted miRNAs, the strongest direct evidence for IKK-subunit targeting comes from extra-UCEC experimental models demonstrated that miR-15a-5p and miR-16-5p directly regulate CHUK/IKKα during human monocyte–macrophage differentiation, using 3′UTR luciferase reporter assays with target-site mutations alongside mRNA and protein-level verification. These two miRNAs also appear among the 14 shared CHUK∩IKBKB candidates in our prediction set (Table 2, Figure 6), suggesting a potential dual regulatory role. Separately showed that miR-148a-3p directly represses IKBKB/IKKβ in human aortic-valve interstitial cells through seedmutant 3′UTR reporter assays, with concordant reductions in IKKβ protein, phospho-p65, and down- stream inflammatory gene expression. As miR-148a-3p likewise falls within our shared CHUK∩IKBKB prediction set, it represents a second mechanistically supported candidate. Importantly, these inter- actions have not yet been validated in endometrial carcinoma; their extrapolation to UCEC therefore remains a testable hypothesis rather than an established mechanism [56,57].

Several microRNAs (miRNAs) have been identified as potential common regulators of both CHUK and IKBKB, including the well-documented tumor-suppressive miRNAs such as miR-15a-5p, miR-16-5p, and miR-497-5p [58,59]. These miRNAs are members of the miR-15/16 family, which is recognized for its involvement in cell cycle arrest, apoptosis induction, and NF-κB inhibition across various cancer types, including gynecologic malignancies [59,60]. Additionally, miR-148a-3p and miR-148b-3p, also predicted to target both CHUK and IKBKB, have been associated with the regulation of immune checkpoint molecules and TGF-β signaling, thereby playing dual roles in tumor suppression and immune modulation [61,62,63]. Notably, miR-424-5p and miR-497-5p have been reported to enhance tumor sensitivity to chemotherapy by targeting pathways such as NF-κB and PI3K/AKT [64,65]. Conversely, the miRNAs predicted for CHUK and IKBKG, specifically miR-4457 and miR-4694-3p, are less well-characterized but may represent novel regulatory miRNAs warranting experimental validation, particularly in the context of inflammation-driven tumor microenvironments where CHUK and IKBKG participate in the regulation of canonical NF-κB signaling.

The integration of TCGA-UCEC miRNA and mRNA expression data substantiated a subset of the predicted regulatory relationships through expression-based evidence. Notably, significant inverse correlations were identified between hsa-miR-625-5p and CHUK, hsa-miR-23a-3p and CHUK, and hsa-miR-148a-3p and IKBKB following FDR correction. While these results enhance the biological relevance of the computational predictions, the relatively modest correlation coefficients necessitate cautious interpretation. Furthermore, no significant inverse correlation was detected for the predicted IKBKG-targeting miRNAs, indicating that experimental validation is essential to confirm direct regulatory effects.

Collectively, these findings indicate that the IKK complex, particularly IKBKG, may play a significant role in the pathobiology of UCEC. Its transcriptional overexpression, correlation with advanced disease stages and poor survival outcomes, qualitative immunohistochemical staining patterns consistent with protein expression, extensive interaction network, and miRNA deregulation collectively underscore IKBKG as a candidate prognostic indicator warranting experimental validation. Conversely, although CHUK and IKBKB are established components of canonical NF-κB signaling, the functional consequences of their altered expression in UCEC remain to be determined.

From a translational standpoint, our findings raise the possibility that targeting the NF-κB/IKK axis, particularly IKBKG, may constitute a novel therapeutic strategy for high-risk UCEC, especially in molecularly defined high-risk UCEC; however, functional pathway activity will need to be established using appropriate protein- and phosphorylation-based biomarkers. Several IKKβ inhibitors and NF-κB antagonists have demonstrated preclinical efficacy in other malignancies, and the potential repurposing of these agents for UCEC merits further investigation. Additionally, the restoration of tumor-suppressive miRNAs may offer a complementary approach to indirectly inhibit IKK activity.

In conclusion, our in silico analyses underscore the pivotal role of IKBKG within the IKK complex in facilitating aggressive phenotypes of UCEC. These findings establish a foundation for further experimental exploration of the IKK/NF-κB axis in UCEC, particularly through direct evaluation of functional pathway activity. It is crucial to recognize that changes in the transcriptional levels of CHUK, IKBKB, and IKBKG do not constitute direct evidence of functional NF-κB pathway activation. The canonical NF-κB signaling pathway is subject to extensive regulation at the post-translational level, involving IKK phosphorylation, IκB degradation, and the phosphorylation and nuclear translocation of NF-κB subunits. Given that these functional markers were not directly evaluated, our results suggest transcriptional and network-level dysregulation of the IKK/NF-κB axis rather than confirming pathway activation.

## 5. Conclusions

In conclusion, this study elucidates the distinct roles of CHUK, IKBKB, and IKBKG in the pathogenesis of UCEC. Notably, IKBKG demonstrates the most significant correlation with poor prognosis, indicating its potential among the three genes examined, suggesting its candidacy as a prognostic indicator pending experimental confirmation. The varied expression and regulation of these canonical NF-κB components underscore the complexity of their involvement in endometrial carcinogenesis and highlight the necessity for further mechanistic and translational research.

## Figures and Tables

**Figure 2:**
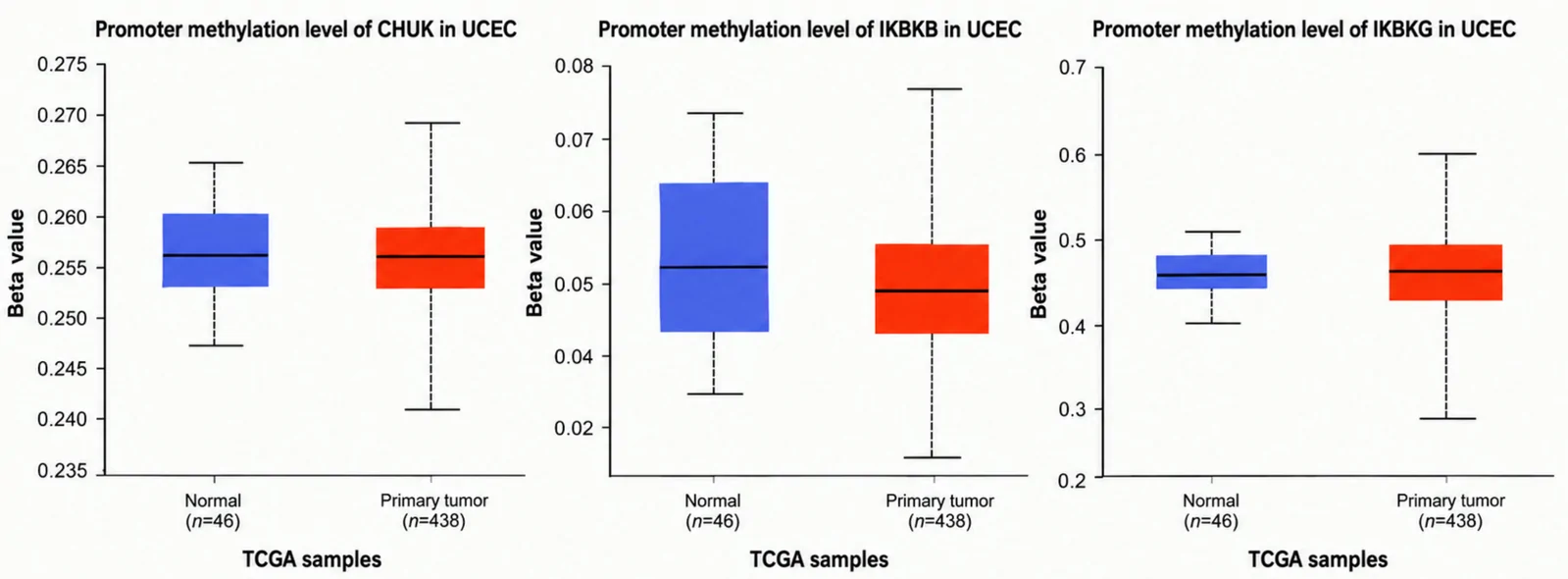
Promoter Methylation Levels of CHUK, IKBKB, and IKBKG in Uterine Corpus Endometrial Carcinoma (UCEC) Based on TCGA Data. Boxplots display the promoter methylation levels (β-values) of CHUK, IKBKB, and IKBKG in normal endometrial tissues (*n* = 46) and primary UCEC tumors (*n* = 438). Promoter methylation analysis revealed gene-specific patterns: CHUK exhibited significant hypomethylation in tumor samples, IKBKB showed no significant methylation difference between tumor and normal tissues, and IKBKG displayed significant hypermethylation in tumors. These group-level comparisons do not establish within-sample methylation expression correlations. Statistical significance was assessed; significant *p*-values are indicated above each comparison. β-values represent the ratio of methylated probe intensity to total signal intensity (methylated + unmethylated), ranging from 0 (unmethylated) to 1 (fully methylated).

**Table 1:** Combined score results of gene–gene interactions.

Gene-1	Gene-2	Protein Annotation	Combined Score
TNFRSF1A	CHUK	Tumor necrosis factor receptor superfamily member 1A	0.999
NFKBIA	CHUK	NF-kappa-B inhibitor alpha	0.999
NFKB1	CHUK	Nuclear factor NF-kappa-B p105 subunit	0.999
IRAK1	CHUK	Interleukin-1 receptor-associated kinase 1	0.999
RELA	CHUK	Transcription factor p65	0.999
TNF	CHUK	Tumor necrosis factor	0.999
IKBKB	CHUK	Inhibitor of nuclear factor kappa-B kinase subunit beta	0.999
TRAF6	CHUK	TNF receptor-associated factor 6	0.999
IKBKG	CHUK	NF-kappa-B essential modulator	0.999
RIPK1	CHUK	Receptor-interacting serine/threonine-protein kinase 1	0.998
TNFRSF1A	IKBKB	Tumor necrosis factor receptor superfamily member 1A	0.999
NFKBIA	IKBKB	NF-kappa-B inhibitor alpha	0.999
NFKB1	IKBKB	Nuclear factor NF-kappa-B p105 subunit	0.999
CHUK	IKBKB	Inhibitor of nuclear factor kappa-B kinase subunit alpha	0.999
RELA	IKBKB	Transcription factor p65	0.999
TNF	IKBKB	Tumor necrosis factor	0.999
TRAF6	IKBKB	TNF receptor-associated factor 6	0.999
IKBKG	IKBKB	NF-kappa-B essential modulator	0.999
NFKBIB	IKBKB	NF-kappa-B inhibitor beta	0.997
KEAP1	IKBKB	Kelch-like ECH-associated protein 1	0.997
TNFRSF1A	IKBKG	Tumor necrosis factor receptor superfamily member 1A	0.999
TAB1	IKBKG	TGF-beta-activated kinase 1 and MAP3K7-binding protein 1	0.999
NFKBIA	IKBKG	NF-kappa-B inhibitor alpha	0.999
NFKB1	IKBKG	Nuclear factor NF-kappa-B p105 subunit	0.998
TRAF2	IKBKG	TNF receptor-associated factor 2	0.999
TANK	IKBKG	TRAF family member-associated NF-kappa-B activator	0.999
RIPK1	IKBKG	Receptor-interacting serine/threonine-protein kinase 1	0.999
RNF31	IKBKG	E3 ubiquitin-protein ligase RNF31	0.999
RBCK1	IKBKG	RanBP-type and C3HC4-type zinc finger-containing protein 1	0.999
ERC1	IKBKG	ELKS/Rab6-interacting/CAST family member 1	0.999

**Table 2:** The miRNAs are associated with CHUK, IKBKB and IKBKG with combination of TargetScanHuman8.0 and miRDB databases.

Predicted miRNAs for CHUK	
miRDB databases TargetScanHuman8.0	hsa-miR-493-5p hsa-miR-340-5p hsa-miR-323a-3p hsa-miR-377-3p hsa-miR-23a-3p hsa-miR-23b-3p hsa-miR-23c hsa-miR-655-3p hsa-miR-374c-5p hsa-miR-6810-5p hsa-miR-625-5p hsa-miR-1275 hsa-miR-4665-5p hsa-miR-29b-2-5p hsa-miR-3136-5p hsa-miR-4439 hsa-miR-6875-3p hsa-miR-4659b-3p hsa-miR-4659a-3p hsa-miR-103b hsa-miR-4652-3p hsa-miR-5680 hsa-miR-3192-3p hsa-miR-22-5p hsa-miR-548o-3p hsa-miR-1323 hsa-miR-888-5p hsa-miR-6866-5p hsa-miR-5699-3p hsa-miR-4421 hsa-miR-4799-5p hsa-miR-4272 hsa-miR-6074 hsa-miR-7-2-3p hsa-miR-7-1-3p hsa-miR-1322 hsa-miR-605-3p hsa-miR-2681-5p hsa-miR-33a-3p hsa-miR-3154 hsa-miR-376c-3p hsa-miR-3117-5p hsa-miR-548at-5p hsa-miR-942-5p hsa-miR-3662 hsa-miR-3173-3p hsa-miR-6891-5p hsa-miR-4457 hsa-miR-4801 hsa-miR-4731-3p hsa-miR-148b-3p hsa-miR-152-3p hsa-miR-148a-3p hsa-miR-4276 hsa-miR-5693 hsa-miR-30a-3p hsa-miR-30e-3p hsa-miR-30d-3p hsa-miR-892c-3p hsa-miR-452-5p hsa-miR-4676-3p hsa-miR-3179 hsa-miR-6770-5p hsa-miR-5003-5p hsa-miR-8485 hsa-miR-126-5p hsa-miR-4795-3p hsa-miR-656-3p hsa-miR-4528 hsa-miR-3671 hsa-miR-607 hsa-miR-1305 hsa-miR-3646 hsa-miR-153-5p hsa-miR-641 hsa-miR-3617-5p hsa-miR-3149 hsa-miR-6759-3p hsa-miR-5683 hsa-miR-6504-3p hsa-miR-4275 hsa-miR-1290 hsa-miR-5004-3p hsa-miR-3942-3p hsa-miR-3688-3p hsa-miR-4760-5p hsa-miR-8061 hsa-miR-4694-3p hsa-miR-224-3p hsa-miR-522-3p hsa-miR-216b-3p hsa-miR-1468-3p hsa-miR-548p hsa-miR-545-3p hsa-miR-16-5p hsa-miR-195-5p hsa-miR-15a-5p hsa-miR-6838-5p hsa-miR-15b-5p hsa-miR-497-5p hsa-miR-424-5p hsa-miR-2113 hsa-miR-5010-3p hsa-miR-4766-5p hsa-miR-146a-3p hsa-miR-374c-3p hsa-miR-4422 hsa-miR-548e-5p hsa-miR-548aj-3p hsa-miR-548x-3p hsa-miR-548j-3p hsa-miR-548ae-3p hsa-miR-548aq-3p hsa-miR-548ah-3p hsa-miR-548am-3p hsa-miR-520h hsa-miR-520g-3p hsa-miR-548h-3p hsa-miR-548d-3p hsa-miR-548ac hsa-miR-548bb-3p hsa-miR-548z hsa-miR-5585-3p hsa-miR-29a-5p
**Predicted miRNAs for IKBKB**	
miRDB databases TargetScanHuman8.0	hsa-miR-199a-5p hsa-miR-199b-5p hsa-miR-15b-5p hsa-miR-424-5p hsa-miR-16-5p hsa-miR-497-5p hsa-miR-195-5p hsa-miR-6838-5p hsa-miR-15a-5p hsa-miR-200c-3p hsa-miR-200b-3p hsa-miR-429 hsa-miR-6798-5p hsa-miR-3189-5p hsa-miR-1913 hsa-miR-4645-5p hsa-miR-4673 hsa-miR-6776-3p hsa-miR-6165 hsa-miR-3619-5p hsa-miR-214-3p hsa-miR-761 hsa-miR-148b-3p hsa-miR-152-3p hsa-miR-148a-3p hsa-miR-4640-5p hsa-miR-4726-5p hsa-miR-7160-5p hsa-miR-6742-3p hsa-miR-4302 hsa-miR-1825 hsa-miR-3925-3p hsa-miR-7154-3p hsa-miR-345-3p hsa-miR-5195-5p hsa-miR-3139 hsa-miR-708-5p hsa-miR-28-5p hsa-miR-6859-5p hsa-miR-3125 hsa-miR-3916 hsa-miR-644a hsa-miR-4766-5p hsa-miR-7976 hsa-miR-874-3p hsa-miR-3173-5p hsa-miR-6799-3p hsa-miR-18a-3p hsa-miR-4768-3p hsa-miR-4722-5p hsa-miR-6510-5p hsa-miR-3120-5p hsa-miR-4519 hsa-miR-4524b-5p hsa-miR-4524a-5p hsa-miR-374b-3p hsa-miR-2116-3p hsa-miR-6513-5p hsa-miR-4291 hsa-miR-3662 hsa-miR-3659 hsa-miR-3130-5p hsa-miR-4482-5p hsa-miR-1284 hsa-miR-3185 hsa-miR-218-5p hsa-miR-1250-3p hsa-miR-330-3p hsa-miR-3942-3p hsa-miR-8087 hsa-miR-1-3p hsa-miR-206 hsa-miR-613 hsa-miR-6753-5p hsa-miR-3156-3p hsa-miR-24-3p hsa-miR-5693
**Predicted miRNAs for IKBKG**	
miRDB databases TargetScanHuman8.0	hsa-miR-518d-3p hsa-miR-518c-3p hsa-miR-518b hsa-miR-939-3p hsa-miR-324-5p hsa-miR-4283 hsa-miR-4267 hsa-miR-4650-5p hsa-miR-1276 hsa-miR-6750-5p hsa-miR-6822-5p hsa-miR-7515 hsa-miR-766-5p hsa-miR-7847-3p hsa-miR-7106-5p hsa-miR-5685 hsa-miR-7156-3p hsa-miR-4694-3p hsa-miR-6504-5p hsa-miR-3064-5p hsa-miR-3912-5p hsa-miR-4457
**Predicted common miRNAs for CHUK vs. IKBKB**	
miRDB databases TargetScanHuman8.0	hsa-miR-3662 hsa-miR-148b-3p hsa-miR-152-3p hsa-miR-148a-3p hsa-miR-5693 hsa-miR-3942-3p hsa-miR-16-5p hsa-miR-195-5p hsa-miR-15a-5p hsa-miR-6838-5p hsa-miR-15b-5p hsa-miR-497-5p hsa-miR-424-5p hsa-miR-4766-5p
**Predicted common miRNAs for CHUK vs. IKBKG**	
miRDB databases TargetScanHuman8.0	hsa-miR-4457 hsa-miR-4694-3p

**Table 3:** Univariate and multivariable Cox proportional hazards regression analysis for overall survival in UCEC.

Variable	HR	95% CI	*p* Value
**Univariate analysis**			
IKBKG high vs. low	**1.62**	**1.04–2.53**	**0.033**
**Multivariable analysis**			
IKBKG high vs. low	**1.58**	**1.00–2.47**	**0.048**
Age, per year	1.00	0.98–1.03	0.793
Non-endometrioid vs. endometrioid histology	1.19	0.67–2.11	0.554
High vs. low histological grade	**3.17**	**1.54–6.52**	**0.002**
CN-high vs. CN-low	1.66	0.75–3.70	0.214
MSI vs. CN-low	1.09	0.51–2.34	0.830
POLE vs. CN-low	**0.19**	**0.04–0.90**	**0.036**

Table Legend: Table 3 presents the results of a univariate and multivariable Cox proportional hazards regression analysis concerning overall survival in patients with UCEC. The expression of IKBKG was dichotomized at the median expression level of 439.297. The multivariable model incorporated variables such as IKBKG expression, age at diagnosis, histological type, histological grade, and TCGA molecular subtype. Histological type was classified into endometrioid and non-endometrioid categories, while histological grade was divided into low grade (G1/G2) and high grade (G3/high grade). The CN-low category served as the reference for the TCGA molecular subtype. Abbreviations: HR, hazard ratio; CI, confidence interval; CN, copy-number; MSI, microsatellite instability.

## Data Availability

All data analyzed in this study were obtained from publicly available databases and do not require additional permissions for access. Transcriptomic expression data were retrieved from the TCGA-UCEC dataset via GEPIA2 (http://gepia2.cancer-pku.cn/, accessed on 7 January 2025) and TIMER 2.0 (http://timer.cistrome.org/). Promoter methylation and clinicopathological subgroup data were obtained from UALCAN (https://ualcan.path.uab.edu/, accessed on 7 January 2025) using TCGA-UCEC Illumina HumanMethylation450 data. Protein expression data were sourced from the Human Protein Atlas (https://www.proteinatlas.org/, version 24.0). Protein–protein interaction data were retrieved from the STRING database (https://string-db.org/, version 12.0). MicroRNA target predictions were obtained from miRDB (https://mirdb.org/) and TargetScan Human 8.0 (https://www.targetscan.org/vert_80/, accessed on 7 January 2025). External validation datasets GSE7305 and GSE25628 were downloaded from the NCBI Gene Expression Omnibus (https://www.ncbi.nlm.nih.gov/geo/, accessed on 7 January 2025). Survival analysis was performed using the Kaplan–Meier Plotter (https://kmplot.com/analysis/, accessed on 7 January 2025). No new datasets were generated in this study.

## Abbreviations

The following abbreviations are used in this manuscript:

UCEC	Uterine Corpus Endometrial Carcinoma
IKK	IκB Kinase
CHUK	Conserved Helix-Loop-Helix Ubiquitous Kinase (also known as IKKα)
IKBKB	Inhibitor of Nuclear Factor Kappa B Kinase Subunit Beta (also known as IKKβ)
IKBKG	Inhibitor of Nuclear Factor Kappa B Kinase Regulatory Subunit Gamma (also known as NEMO)
NF-κB	Nuclear Factor kappa-light-chain-enhancer of activated B cells
EMT	Epithelial-to-Mesenchymal Transition
PPI	Protein–Protein Interaction
miRNA	MicroRNA
HPA	Human Protein Atlas
STRING	Search Tool for the Retrieval of Interacting Genes/Proteins
TCGA	The Cancer Genome Atlas
GEPIA2	Gene Expression Profiling Interactive Analysis 2
TIMER2.0	Tumor Immune Estimation Resource 2.0
RNA-seq	RNA Sequencing
FC	Fold Change
log_2_FC	Log base 2 Fold Change
FDR	False Discovery Rate
TPM	Transcripts Per Million
KEGG	Kyoto Encyclopedia of Genes and Genomes
GO	Gene Ontology
IL	Interleukin
TLR	Toll-like Receptor
TNF	Tumor Necrosis Factor
STAT3	Signal Transducer and Activator of Transcription 3
MAPK	Mitogen-Activated Protein Kinase
PI3K/AKT	Phosphatidylinositol 3-Kinase/Protein Kinase B pathway
H&E	Hematoxylin and Eosin
OS	Overall Survival

## References

[1] Doll K.M., Winn A.N. (2019). Assessing Endometrial Cancer Risk among US Women: Long-Term Trends Using Hysterectomy-Adjusted Analysis. Am. J. Obstet. Gynecol..

[2] Zheng Q.H., Shi L., Li H.L. (2019). FALEC Exerts Oncogenic Properties to Regulate Cell Proliferation and Cell-Cycle in Endometrial Cancer. Biomed. Pharmacother..

[3] Smrz S.A., Calo C., Fisher J.L., Salani R. (2021). An Ecological Evaluation of the Increasing Incidence of Endometrial Cancer and the Obesity Epidemic. Am. J. Obstet. Gynecol..

[4] Felix A.S., Weissfeld J.L., Stone R.A., Bowser R., Chivukula M., Edwards R.P., Linkov F. (2010). Factors Associated with Type i and Type II Endometrial Cancer. Cancer Causes Control.

[5] Raglan O., Kalliala I., Markozannes G., Cividini S., Gunter M.J., Nautiyal J., Gabra H., Paraskevaidis E., Martin-Hirsch P., Tsilidis K.K. (2019). Risk Factors for Endometrial Cancer: An Umbrella Review of the Literature. Int. J. Cancer.

[6] Diéguez-Martínez N., Espinosa-Gil S., Yoldi G., Megías-Roda E., Bolinaga-Ayala I., Viñas-Casas M., Gorgisen G., Domingo-Ortí I., Pérez-Montoyo H., Bayascas J.R. (2022). The ERK5/NF-ΚB Signaling Pathway Targets Endometrial Cancer Proliferation and Survival. Cell. Mol. Life Sci..

[7] Yu H., Lin L., Zhang Z., Zhang H., Hu H. (2020). Targeting NF-ΚB Pathway for the Therapy of Diseases: Mechanism and Clinical Study. Signal Transduct. Target. Ther..

[8] Pallares J., Martínez-Guitarte J.L., Dolcet X., Llobet D., Rue M., Palacios J., Prat J., Matias-Guiu X. (2004). Abnormalities in the NF-ΚB Family and Related Proteins in Endometrial Carcinoma. J. Pathol..

[9] Dong P., Kaneuchi M., Watari H., Hamada J., Sudo S., Ju J., Sakuragi N. (2011). MicroRNA-194 Inhibits Epithelial to Mesenchymal Transition of Endometrial Cancer Cells by Targeting Oncogene BMI-1. Mol. Cancer.

[10] Shin J., Lim J., Han D., Lee S., Sung N.S., Kim J.S., Kim D.K., Lee H.Y., Lee S.K., Shin J. (2025). TBK1 Inhibitor Amlexanox Exerts Anti-Cancer Effects against Endometrial Cancer by Regulating AKT/NF-ΚB Signaling. Int. J. Biol. Sci..

[11] Żebrowska-Nawrocka M., Szmajda-Krygier D., Krygier A., Jeleń A., Balcerczak E. (2024). Bioinformatic Analysis of IKK Complex Genes Expression in Selected Gastrointestinal Cancers. Int. J. Mol. Sci..

[12] Oeckinghaus A., Ghosh S. (2009). The NF-KappaB Family of Transcription Factors and Its Regulation. Cold Spring Harb. Perspect. Biol..

[13] Boehm J.S., Zhao J.J., Yao J., Kim S.Y., Firestein R., Dunn I.F., Sjostrom S.K., Garraway L.A., Weremowicz S., Richardson A.L. (2007). Integrative Genomic Approaches Identify IKBKE as a Breast Cancer Oncogene. Cell.

[14] Wang J., Kong X. (2025). IKBKE Modulates Autophagy and Progestin Resistance in Endometrial Cancer. Front. Oncol..

[15] Chau T.L., Gioia R., Gatot J.S., Patrascu F., Carpentier I., Chapelle J.P., O’Neill L., Beyaert R., Piette J., Chariot A. (2008). Are the IKKs and IKK-Related Kinases TBK1 and IKK-ε Similarly Activated?. Trends Biochem. Sci..

[16] Tang Z., Kang B., Li C., Chen T., Zhang Z. (2019). GEPIA2: An Enhanced Web Server for Large-Scale Expression Profiling and Interactive Analysis. Nucleic Acids Res..

[17] Chandrashekar D.S., Karthikeyan S.K., Korla P.K., Patel H., Shovon A.R., Athar M., Netto G.J., Qin Z.S., Kumar S., Manne U. (2022). UALCAN: An Update to the Integrated Cancer Data Analysis Platform. Neoplasia.

[18] Li T., Fu J., Zeng Z., Cohen D., Li J., Chen Q., Li B., Liu X.S. (2020). TIMER2.0 for Analysis of Tumor-Infiltrating Immune Cells. Nucleic Acids Res..

[19] Szklarczyk D., Kirsch R., Koutrouli M., Nastou K., Mehryary F., Hachilif R., Gable A.L., Fang T., Doncheva N.T., Pyysalo S. (2023). The STRING Database in 2023: Protein-Protein Association Networks and Functional Enrichment Analyses for Any Sequenced Genome of Interest. Nucleic Acids Res..

[20] Chen Y., Wang X. (2020). MiRDB: An Online Database for Prediction of Functional MicroRNA Targets. Nucleic Acids Res..

[21] McGeary S.E., Lin K.S., Shi C.Y., Pham T.M., Bisaria N., Kelley G.M., Bartel D.P. (2019). The Biochemical Basis of MicroRNA Targeting Efficacy. Science.

[22] Agarwal V., Bell G.W., Nam J.W., Bartel D.P. (2015). Predicting Effective MicroRNA Target Sites in Mammalian MRNAs. eLife.

[23] Hever A., Roth R.B., Hevezi P., Marin M.E., Acosta J.A., Acosta H., Rojas J., Herrera R., Grigoriadis D., White E. (2007). Human Endometriosis Is Associated with Plasma Cells and Overexpression of B Lymphocyte Stimulator. Proc. Natl. Acad. Sci. USA.

[24] Crispi S., Piccolo M.T., D’avino A., Donizetti A., Viceconte R., Spyrou M., Calogero R.A., Baldi A., Signorile P.G. (2013). Transcriptional Profiling of Endometriosis Tissues Identifies Genes Related to Organogenesis Defects. J. Cell. Physiol..

[25] Dou Y., Kawaler E.A., Cui Zhou D., Gritsenko M.A., Huang C., Blumenberg L., Karpova A., Petyuk V.A., Savage S.R., Satpathy S. (2020). Proteogenomic Characterization of Endometrial Carcinoma. Cell.

[26] Zhang Y., Parmigiani G., Johnson W.E. (2020). ComBat-Seq: Batch Effect Adjustment for RNA-Seq Count Data. NAR Genom. Bioinform..

[27] Thompson J.A., Tan J., Greene C.S. (2016). Cross-Platform Normalization of Microarray and RNA-Seq Data for Machine Learning Applications. PeerJ.

[28] Ritchie M.E., Phipson B., Wu D., Hu Y., Law C.W., Shi W., Smyth G.K. (2015). Limma Powers Differential Expression Analyses for RNA-Sequencing and Microarray Studies. Nucleic Acids Res..

[29] Gyorffy B., Surowiak P., Budczies J., Lánczky A. (2013). Online Survival Analysis Software to Assess the Prognostic Value of Biomarkers Using Transcriptomic Data in Non-Small-Cell Lung Cancer. PLoS ONE.

[30] Karin M. (2009). NF-KappaB as a Critical Link between Inflammation and Cancer. Cold Spring Harb. Perspect. Biol..

[31] Hayden M.S., Ghosh S. (2011). NF-ΚB in Immunobiology. Cell Res..

[32] Xia L., Tan S., Zhou Y., Lin J., Wang H., Oyang L., Tian Y., Liu L., Su M., Wang H. (2018). Role of the NFκB-Signaling Pathway in Cancer. Onco Targets Ther..

[33] Perkins N.D. (2012). The Diverse and Complex Roles of NF-ΚB Subunits in Cancer. Nat. Rev. Cancer.

[34] Sun S.C. (2017). The Non-Canonical NF-ΚB Pathway in Immunity and Inflammation. Nat. Rev. Immunol..

[35] Grivennikov S.I., Greten F.R., Karin M. (2010). Immunity, Inflammation, and Cancer. Cell.

[36] Sanchez-Vega F., Mina M., Armenia J., Chatila W.K., Luna A., La K.C., Dimitriadoy S., Liu D.L., Kantheti H.S., Saghafinia S. (2018). Oncogenic Signaling Pathways in The Cancer Genome Atlas. Cell.

[37] Chen L., Deng H., Cui H., Fang J., Zuo Z., Deng J., Li Y., Wang X., Zhao L. (2018). Inflammatory Responses and Inflammation-Associated Diseases in Organs. Oncotarget.

[38] Yu H., Pardoll D., Jove R. (2009). STATs in Cancer Inflammation and Immunity: A Leading Role for STAT3. Nat. Rev. Cancer.

[39] Jones P.A., Baylin S.B. (2007). The Epigenomics of Cancer. Cell.

[40] Cornel K.M.C., Wouters K., Van de Vijver K.K., van der Wurff A.A.M., van Engeland M., Kruitwagen R.F.P.M., Pijnenborg J.M.A. (2019). Gene Promoter Methylation in Endometrial Carcinogenesis. Pathol. Oncol. Res..

[41] Trimarchi M.P., Yan P., Groden J., Bundschuh R., Goodfellow P.J. (2017). Identification of Endometrial Cancer Methylation Features Using Combined Methylation Analysis Methods. PLoS ONE.

[42] Irizarry R.A., Bolstad B.M., Collin F., Cope L.M., Hobbs B., Speed T.P. (2003). Summaries of Affymetrix GeneChip Probe Level Data. Nucleic Acids Res..

[43] Shi L., Reid L.H., Jones W.D., Shippy R., Warrington J.A., Baker S.C., Collins P.J., De Longueville F., Kawasaki E.S., Lee K.Y. (2006). The MicroArray Quality Control (MAQC) Project Shows Inter- and Intraplatform Reproducibility of Gene Expression Measurements. Nat. Biotechnol..

[44] Wang Z., Gerstein M., Snyder M. (2009). RNA-Seq: A Revolutionary Tool for Transcriptomics. Nat. Rev. Genet..

[45] Conesa A., Madrigal P., Tarazona S., Gomez-Cabrero D., Cervera A., McPherson A., Szcześniak M.W., Gaffney D.J., Elo L.L., Zhang X. (2016). A Survey of Best Practices for RNA-Seq Data Analysis. Genome Biol..

[46] Hu R., Islam M.N., Varghese R.S., Ressom H.W. (2024). Short-Read RNA-Seq. Methods in Molecular Biology.

[47] Marioni J.C., Mason C.E., Mane S.M., Stephens M., Gilad Y. (2008). RNA-Seq: An Assessment of Technical Reproducibility and Comparison with Gene Expression Arrays. Genome Res..

[48] Su Z., Łabaj P.P., Li S., Thierry-Mieg J., Thierry-Mieg D., Shi W., Wang C., Schroth G.P., Setterquist R.A., Thompson J.F. (2014). A Comprehensive Assessment of RNA-Seq Accuracy, Reproducibility and Information Content by the Sequencing Quality Control Consortium. Nat. Biotechnol..

[49] Zhao S., Fung-Leung W.P., Bittner A., Ngo K., Liu X. (2014). Comparison of RNA-Seq and Microarray in Transcriptome Profiling of Activated T Cells. PLoS ONE.

[50] Leek J.T., Scharpf R.B., Bravo H.C., Simcha D., Langmead B., Johnson W.E., Geman D., Baggerly K., Irizarry R.A. (2010). Tackling the Widespread and Critical Impact of Batch Effects in High-Throughput Data. Nat. Rev. Genet..

[51] O’Mara T.A., Zhao M., Spurdle A.B. (2016). Meta-Analysis of Gene Expression Studies in Endometrial Cancer Identifies Gene Expression Profiles Associated with Aggressive Disease and Patient Outcome. Sci. Rep..

[52] Li Y., Hong Y., Shen H., Zhou J., Cesar D., Eleutério J., Matsuura M., Liu Y., Luo C., Li Q. (2025). FXR Activation Suppresses NF-ΚB Signaling, Proliferation and Migration in Cervical Cancer Cells. Transl. Cancer Res..

[53] Harrington B.S., Annunziata C.M. (2019). Nf-Κb Signaling in Ovarian Cancer. Cancers.

[54] Liu P., Wang C., Ma C., Wu Q., Zhang W., Lao G. (2016). MicroRNA-23a Regulates Epithelial-to-Mesenchymal Transition in Endometrial Endometrioid Adenocarcinoma by Targeting SMAD3. Cancer Cell Int..

[55] Xie W., Qin W., Kang Y., Zhou Z., Qin A. (2016). MicroRNA-340 Inhibits Tumor Cell Proliferation and Induces Apoptosis in Endometrial Carcinoma Cell Line RL 95-2. Med. Sci. Monit..

[56] Li T., Morgan M.J., Choksi S., Zhang Y., Kim Y.S., Liu Z.G. (2010). MicroRNAs Modulate the Noncanonical Transcription Factor NF-ΚB Pathway by Regulating Expression of the Kinase IKKα during Macrophage Differentiation. Nat. Immunol..

[57] Patel V., Carrion K., Hollands A., Hinton A., Gallegos T., Dyo J., Sasik R., Leire E., Hardiman G., Mohamed S.A. (2015). The Stretch Responsive MicroRNA MiR-148a-3p Is a Novel Repressor of IKBKB, NF-ΚB Signaling, and Inflammatory Gene Expression in Human Aortic Valve Cells. FASEB J..

[58] Liu C., Bordeaux A., Hettich S., Han S. (2020). MicroRNA-497-5p Functions as a Modulator of Apoptosis by Regulating Metadherin in Ovarian Cancer. Cell Transplant..

[59] Aqeilan R.I., Calin G.A., Croce C.M. (2010). MiR-15a and MiR-16-1 in Cancer: Discovery, Function and Future Perspectives. Cell Death Differ..

[60] Calin G.A., Cimmino A., Fabbri M., Ferracin M., Wojcik S.E., Shimizu M., Taccioli C., Zanesi N., Garzon R., Aqeilan R.I. (2008). MiR-15a and MiR-16-1 Cluster Functions in Human Leukemia. Proc. Natl. Acad. Sci. USA.

[61] Zheng B., Liang L., Wang C., Huang S., Cao X., Zha R., Liu L., Jia D., Tian Q., Wu J. (2011). MicroRNA-148a Suppresses Tumor Cell Invasion and Metastasis by Downregulating ROCK1 in Gastric Cancer. Clin. Cancer Res..

[62] Klicka K., Grzywa T.M., Klinke A., Mielniczuk A., Włodarski P.K. (2021). The Role of Mirnas in the Regulation of Endometrial Cancer Invasiveness and Metastasis—A Systematic Review. Cancers.

[63] Chen R., Ma X., Zhang L. (2020). MicorRNA-148b Inhibits Cell Proliferation and Facilitates Cell Apoptosis by RegulatingDNA Methyltransferase 1 in Endometrial Cancer. Transl. Cancer Res..

[64] Li S., Wu Y., Zhang J., Sun H., Wang X. (2020). Role of Mirna-424 in Cancers. Onco Targets. Ther..

[65] Cheng D., Zhu C., Liang Y., Xing Y., Shi C. (2020). MiR-424 Overexpression Protects Alveolar Epithelial Cells from LPS-Induced Apoptosis and Inflammation by Targeting FGF2 via the NF-ΚB Pathway. Life Sci..

